# Considerations in adapting CRISPR/Cas9 in nongenetic model plant systems

**DOI:** 10.1002/aps3.11314

**Published:** 2020-01-12

**Authors:** Shengchen Shan, Pamela S. Soltis, Douglas E. Soltis, Bing Yang

**Affiliations:** ^1^ Plant Molecular and Cellular Biology Program University of Florida Gainesville Florida 32611‐0180 USA; ^2^ Florida Museum of Natural History University of Florida Gainesville Florida 32611‐7800 USA; ^3^ Biodiversity Institute University of Florida Gainesville Florida 32611‐5585 USA; ^4^ Genetics Institute University of Florida Gainesville Florida 32610 USA; ^5^ Department of Biology University of Florida Gainesville Florida 32611‐8525 USA; ^6^ Division of Plant Sciences University of Missouri Columbia Missouri 65211 USA; ^7^ Donald Danforth Plant Science Center St. Louis Missouri 63132 USA

**Keywords:** CRISPR/Cas9, genome editing, nongenetic model

## Abstract

The past six years have seen the rapid growth of studies of CRISPR/Cas9 in plant genome editing, a method that enormously facilitates both basic research and practical applications. Most studies have focused on genetic model species, but plant species that are not genetic models may also be economically important or biologically significant, or both. However, developing the CRISPR/Cas9 system in a nongenetic model is challenging. Here, we summarize CRISPR/Cas9 applications in 45 plant genera across 24 families and provide a reference for practical application of CRISPR in nongenetic model plant systems. Suggestions for selecting plant species and target genes are given for proof‐of‐principle CRISPR studies, and the processes of vector construction are reviewed. We recommend using transient assays to identify a desired CRISPR/Cas9 system in a nongenetic model. We then review methods of plant transformation and describe approaches, using regenerated transgenic plants, for evaluating CRISPR editing results. Lastly, potential future applications of CRISPR in nongenetic model plant species are discussed. This review provides a road map for developing CRISPR in nongenetic models, an application that holds enormous potential in plant biology.

The rapid development and application of the CRISPR system in genome editing and other applications clearly illustrate its revolutionary role in biological research. Its massive impact is similar to that of molecular cloning and PCR technologies (Yin et al., 2017). The CRISPR (clustered regularly interspaced short palindromic repeats)–Cas (CRISPR‐associated) (see Box 1 for all abbreviations used in this article) system originated from the microbe immune system and was adapted to provide powerful tools to enable genome editing (Belhaj et al., 2015; Lander, 2016). The constantly expanding CRISPR toolbox comprises various Cas proteins (e.g., Cas9, Cas12a, and Cas13) and their engineered variants, as well as orthologs from diverse bacterial species (Zhang et al., 2019a). In addition to genome editing, CRISPR technology has also been widely applied in transcriptome regulation and epigenome editing (Zhang et al., 2019a). Among various CRISPR systems, engineered class 2 CRISPR/Cas9 is the most popular and robust, especially the Cas9 from *Streptococcus pyogenes* (SpCas9). Therefore, unless otherwise noted, this review is focused on studies of CRISPR/SpCas9, shortened as CRISPR/Cas9, in plant genome editing.

Box 1 **Abbreviations used in this article.**

**Table:** 

Abbreviation	Description
ABCE model	The ABCE model specifies the genetic basis of floral‐organ identity: sepal identity is conferred by class A genes; class A and B activity together specify petal identity; stamen identity is conferred by class B and C genes; class C activity specifies carpel identity; class E genes interact with A, B, and C genes to specify floral‐organ identity
Cas	CRISPR‐associated
CRISPR	clustered regularly interspaced short palindromic repeats
crRNA	CRISPR RNA
DSB	double‐stranded DNA break
FLAG	sequence motif DYKDDDDK (where D = aspartic acid, Y = tyrosine, and K = lysine)
HDR	homology‐directed repair
indel	insertion or deletion
NHEJ	non‐homologous end joining
NLS	nuclear localization signal
PAM	protospacer adjacent motif
sgRNA	single‐guide RNA
snRNA	small nuclear RNA
spCas9	Cas9 from *Streptococcus pyogenes*
TALENs	transcription‐activator‐like effector nucleases
tracrRNA	trans‐activating crRNA
U3/U6 promoter	*U3* or *U6* small nuclear RNA gene promoter
WGD	whole‐genome duplication
ZFNs	zinc‐finger nucleases

There are two components of the CRISPR/Cas9 system: the Cas9 endonuclease and the single‐guide RNA (sgRNA) (Fig. 1). The ribonucleoprotein Cas9‐sgRNA complex recognizes and binds any genomic regions that contain a protospacer adjacent motif (PAM) sequence, which is NGG (where N represents any nucleotide) for SpCas9. If the spacer sequence of the sgRNA (i.e., the first 20 nucleotides at its 5′ end; Fig. 1) matches the genomic sequence immediately upstream of the PAM sequence, Cas9 will cleave both strands of the genomic DNA, leaving blunt ends at the position between the third and fourth nucleotides upstream of PAM (Fig. 1; Jinek et al., 2012). The double‐stranded DNA break (DSB) will be repaired by one of the two innate DNA repair systems: the non‐homologous end‐joining (NHEJ) pathway or homology‐directed repair (HDR) pathway (Symington and Gautier, 2011). The error‐prone NHEJ pathway is efficient and could introduce a small insertion or deletion (indel) at the DSB point (Fig. 1). When occurring in a gene‐coding region, the indel might lead to a frameshift mutation or a premature stop codon in the target gene, and this approach has been widely used for gene knockouts (reviewed in Karkute et al., 2017; Bewg et al., 2018; Modrzejewski et al., 2019; Zhang et al., 2019a). In addition, the occurrence of a CRISPR‐mediated indel in the promoter of a gene might interfere with transcription factor binding and alter gene expression (reviewed in Langner et al., 2018). Compared to the NHEJ pathway, the HDR pathway is less efficient but more accurate. In the presence of a DNA template, either single‐ or double‐stranded, the resultant DNA sequence will be the same as the template, which has been used for gene replacement or targeted insertion (Fig. 1; reviewed in Scheben et al., 2017). Because NHEJ is the dominant pathway for DNA repair, HDR‐mediated gene replacement is challenging in plants (Scheben et al., 2017); on the other hand, approaches improving HDR have been reported (Zhang et al., 2019a).

**Figure 1:**
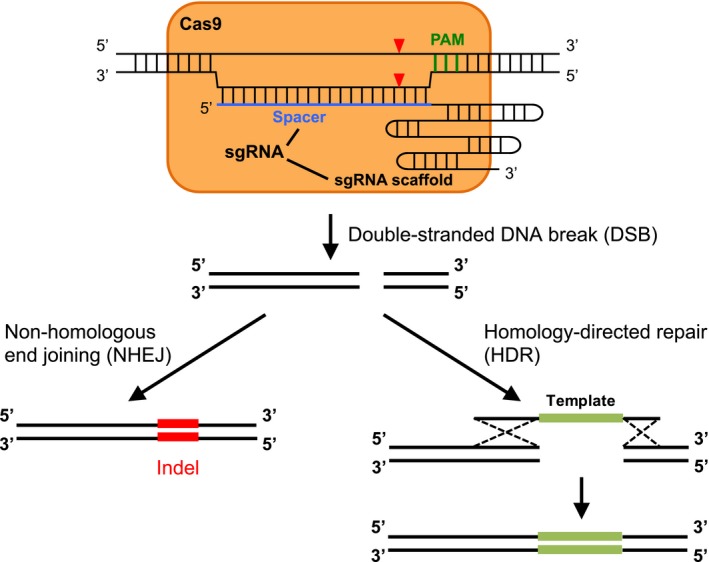
Schematic description of the mechanisms of CRISPR/Cas9‐induced genome editing. The Cas9‐sgRNA complex binds any genomic region with a PAM sequence (shown in green). If the spacer sequence (the first 20 nucleotides at the 5′ end, shown in blue) of the sgRNA is complementary to the genomic sequence immediately upstream of PAM, the Cas9 endonuclease will make a DSB at three nucleotides upstream of PAM (indicated by red triangles). If the DSB is repaired by the error‐prone NHEJ pathway, an indel could be introduced at the DSB site. An indel within an exon or a gene promoter knocks out the gene of interest. Alternatively, with the presence of a donor template (single‐ or double‐stranded), which is flanked by sequences that are homologous to the genomic region adjacent to the DSB (indicated by the dotted lines), gene replacement can be introduced through the HDR pathway.

As the most recent and advanced approach in targeted genome editing, CRISPR has advantages over zinc‐finger nucleases (ZFNs) and transcription‐activator‐like effector nucleases (TALENs) (Sander and Joung, 2014; Shan et al., 2014). ZFNs and TALENs use customized zinc‐finger proteins and transcription‐activator‐like effector proteins for target DNA recognition, respectively. Therefore, both ZFNs and TALENs require complicated processes of protein design and engineering. The modularly assembled repeats of zinc finger and transcription‐activator‐like effector proteins are then fused with the DNA cleavage domain of the FokI endonuclease, resulting in ZFN or TALEN, respectively. Because FokI requires dimerization for its nucleolytic activity, ZFNs and TALENs are engineered in pairs to generate DSBs at the genomic loci of interest. Because of its simplicity, the delivery of CRISPR/Cas9 components into cells is easier than delivery of ZFN/TALEN components. In addition, the simple and programmable features of sgRNAs make CRISPR‐mediated multiplex gene editing possible (Campa et al., 2019), which is unimaginable for ZFNs and TALENs.

Despite their ease of use and potency in activity, CRISPR systems have certain limitations. First, albeit at a low frequency, off‐target (unintended loci with shared sequence similarity) effects of CRISPR/Cas9 in plants have been reported (mutations are detected in ~3% of analyzed potential off‐target sites; reviewed in Modrzejewski et al., 2019). Nevertheless, compared with gene therapy in humans, off‐target effects are less problematic in plant genome editing because off‐target mutants can be discarded and the undesired mutations can be eliminated through backcrossing (Belhaj et al., 2015). In addition, Cas9 variants with enhanced editing specificity have been reported, including eSpCas9 (Slaymaker et al., 2016), xCas9 (Hu et al., 2018a), HypaCas9 (Chen et al., 2017), and paired nCas9s (Ran et al., 2013). Second, a required PAM sequence restricts the positions at which the Cas9‐sgRNA complex can bind. On the other hand, alternative PAM sequences from Cas9 and Cas12a variants and orthologs expand CRISPR target sites, such as NG PAM for SpCas9‐NG (Nishimasu et al., 2018), NGA PAM for SpCas9 VQR variant (Kleinstiver et al., 2015), and TTTV (V can be A, C, or G) PAM for AsCas12a (Cas12a from *Acidaminococcus* sp. BV3L6) (Zetsche et al., 2015).

The engineered CRISPR/Cas9 was first used for genome editing in human and mouse cells in 2013 (Cong et al., 2013; Mali et al., 2013). Soon after, CRISPR/Cas9 was adopted in plant model species, including *Arabidopsis* Heynh., *Oryza* L., and *Nicotiana* L. (e.g., Jiang et al., 2013; Shan et al., 2013; Xie and Yang, 2013). To our knowledge, by 2019, CRISPR has been established and applied across 45 genera from 24 families in land plants, including *Arabidopsis*, major crops, and several ornamental and medicinal plants (Fig. 2, Appendix S1). The most widely studied angiosperm families (in terms of number of genera with established CRISPR systems) are Poaceae, Asteraceae, Solanaceae, Brassicaceae, and Fabaceae.

**Figure 2:**
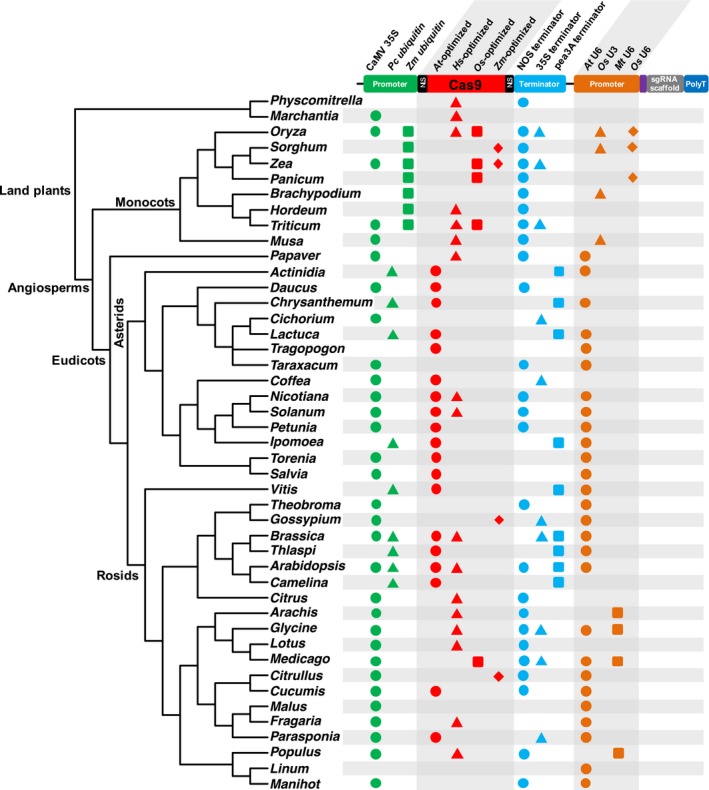
Summary phylogeny of land plant genera with established CRISPR systems; each genus is followed by the CRISPR vector components employed in that system. A full list of references and construct information can be found in Appendix S1. For each component of the vector (e.g., the promoter that drives *Cas9*), the most commonly used items are listed from left to right using different shapes (circle, triangle, square, and diamond) to represent different items. Promoters that drive *Cas9*: CaMV *35S* = *Cauliflower mosaic virus 35S* promoter; *Pc ubiquitin* = *Petroselinum crispum ubiquitin* promoter; *Zm ubiquitin* = *Zea mays ubiquitin* promoter. The most widely used codon‐optimized *Cas9*s are for *Arabidopsis thaliana*,* Homo sapiens*,* Oryza sativa*, and *Z. mays*; either single or dual NLS is fused with *Cas9*. Terminators following *Cas9*: NOS terminator = termination sequence of the nopaline synthase gene; 35S terminator = 35S terminator from CaMV; pea3A terminator = *Pisum sativum* pea3A terminator. *At* U6, *Os* U6, and *Mt* U6 represent *U6* snRNA gene promoters from *A. thaliana*,* O. sativa,* and *Medicago truncatula,* respectively. *Os* U3 represents *U3* snRNA gene promoter from *O. sativa*. The phylogenetic relationships among genera is retrieved from the Open Tree of Life project using the R package *rotl* (Michonneau et al., 2016).

CRISPR‐mediated genome‐edited plants have shown increased yield and growth characteristics, improved food and feed quality, increased resistance to biotic and abiotic stresses, and better herbicide tolerance and industrial utilization (reviewed in Modrzejewski et al., 2019). For instance, in *Gossypium* L., Wang et al. (2017) knocked out the arginase gene using CRISPR/Cas9, and the mutants had improved lateral root formation; in *Oryza*, CRISPR‐mediated loss of function of *OsSK41* resulted in increased grain length and weight (Hu et al., 2018b); in *Camelina* Crantz, seed lipid profiles have been changed by targeted mutagenesis of *FAD2* genes (Morineau et al., 2017); by knocking out *4′OMT2*, biosynthesis of alkaloids was altered in *Papaver* L. leaves (Alagoz et al., 2016); in *Vitis* L., knocking out the *VvWRKY52* transcription factor gene increased fungal resistance (Wang et al., 2018); genome‐edited tomato (*Solanum lycopersicum* L.) with long shelf life was generated by CRISPR/Cas9 through replacing the *ALC* allele by the *alc* allele via the HDR repair pathway (Yu et al., 2017); in *Citrus* L., CRISPR‐mediated modification of the TAL effector binding element of *CsLOB1* gene promoter generated canker‐resistant plants (Peng et al., 2017); by using a Cas9 variant (BE3), CRISPR‐mediated base‐editing in the *ALS* gene produced edited watermelon (*Citrullus lanatus* (Thunb.) Matsum. & Nakai) with herbicide resistance (Tian et al., 2018); in *Populus* L., the *4CL1* gene was knocked out by CRISPR, and the mutants showed decreased lignin content (Zhou et al., 2015).

A phylogenetic summary tree, denoting plant systems with their established CRISPR system (Fig. 2), provides not only an overview of CRISPR applications in plants, but also a reference for researchers who want to develop the CRISPR platform in new plant systems. The process of developing CRISPR from a closely related species can be adapted for an initial attempt of establishing CRISPR in a newly studied plant species.

We define a nongenetic model plant system as a species that does not have a reference genome and a de novo established plant transformation system, but provides a unique study system because of either economic importance or research significance, or both. To our knowledge, CRISPR‐mediated knockout of the phytoene desaturase gene (*TraPDS*) in diploid and tetraploid *Tragopogon* L. (Asteraceae) represents the first CRISPR study in a nongenetic model (Shan et al., 2018). CRISPR application in *Tragopogon* polyploids from natural populations will enable an unprecedented opportunity to study the genetic consequences immediately following polyploidy, a major evolutionary force in plants (Soltis and Soltis, 2009; Van de Peer et al., 2017; Landis et al., 2018). In addition, an established genome editing system in *Tragopogon* facilitates functional biology research on important developmental processes, including the formation of the inflorescences unique to Asteraceae.

In this review, based on our experience in developing a CRISPR system in *Tragopogon* (Shan et al., 2018), we provide a step‐by‐step guide of how to establish CRISPR in nongenetic model plants by summarizing CRISPR studies from various plant systems. Given the significance of nongenetic model plants in both basic and applied research, CRISPR applications in those systems have enormous potential in plant biology.

## CHOICE OF PLANT SPECIES

In developing a CRISPR system in nongenetic models, not all plant species will exhibit the same challenges. Based on our survey of CRISPR applications in plants, most species are herbaceous annuals or biennials with a highly homozygous diploid genome. Establishing a CRISPR system in perennials and polyploids is more challenging than in related diploids. Therefore, when choosing a nongenetic model plant system in a proof‐of‐principle CRISPR study, ploidal level, genome heterozygosity, growth cycle, and physiological characters of the species need to be considered.

A high ploidal level leads to an increased workload for CRISPR to edit all copies of the target gene. If a wide range of ploidal levels is present in the species or genus of interest, we suggest that investigators first develop the CRISPR system in a diploid representative. In our study system, *Tragopogon*, there are both diploid and tetraploid species. To identify a workable CRISPR system, we first demonstrated the genome editing ability of CRISPR in diploid (2*n* = 12) *T. porrifolius* L. before we edited the same target gene in its allopolyploid derivative *T. mirus* G. B. Ownbey (2*n* = 24) (Shan et al., 2018). As expected, the editing efficiency of CRISPR is higher in the diploid parental species (87%) compared to the allopolyploid (78%) (Shan et al., 2018). To knock out all homeologs of the target gene in an allopolyploid (or all alleles in an outcrossing species with high heterozygosity), sgRNA(s) could be designed to target the conserved sequences of different homeologs/alleles (e.g., Zhou et al., 2015; Morineau et al., 2017; Wang et al., 2017; Liu et al., 2018; Shan et al., 2018; Yuan et al., 2019). Even with a relatively low genome editing efficiency, CRISPR has been successfully developed in a few polyploid species, including octoploid strawberry (*Fragaria × ananassa* Duchesne ex Rozier) (Martín‐Pizarro et al., 2018; Wilson et al., 2019); hexaploid wheat (*Triticum aestivum* L.) (Wang et al., 2014; Zhang et al., 2019b) and false flax (*Camelina sativa* (L.) Crantz) (Morineau et al., 2017); tetraploid peanut (*Arachis hypogaea* L.) (Yuan et al., 2019), oilseed rape (*Brassica napus* L.) (Braatz et al., 2017), switchgrass (*Panicum virgatum* L.) (Liu et al., 2018), potato (*Solanum tuberosum* L.) (Andersson et al., 2017), cotton (*Gossypium hirsutum* L.) (Gao et al., 2017; Li et al., 2017b), and *Tragopogon mirus* (Shan et al., 2018); and triploid banana (*Musa* L.) (Kaur et al., 2018). In sum, ploidal level should be a consideration when selecting a nongenetic model plant species, but based on recent studies, CRISPR works well in polyploids.

The growth cycle and physiology of a plant species should also be considered when developing a workable CRISPR system in nongenetic models. These two characters are closely related: shorter‐lived annuals and biennials are mostly herbaceous, and many perennials are woody. Compared with the process in herbaceous plants, because of the long generation time, developing a CRISPR system in woody perennials would encounter more challenges in terms of phenotyping of certain mutants (e.g., flowering traits and seed characters) and evaluating the inheritance of mutated alleles in subsequent generations (Bewg et al., 2018); the outcrossing and/or dioecious nature of many trees adds further challenges for applying CRISPR technology (Bewg et al., 2018). So far, nine of 45 genera with established CRISPR systems contain woody perennials: *Actinidia* Lindl. (Varkonyi‐Gasic et al., 2019), *Citrus* (Jia et al., 2017), *Coffea* L. (Breitler et al., 2018), *Malus* Mill. (Nishitani et al., 2016; Osakabe et al., 2018), *Manihot* Mill. (Odipio et al., 2017), *Parasponia* Miq. (Van Zeijl et al., 2018), *Populus* (Fan et al., 2015; Zhou et al., 2015), *Theobroma* L. (Fister et al., 2018), and *Vitis* (Nakajima et al., 2017; Osakabe et al., 2018; Wang et al., 2018). Most of these studies of woody plants examine mutants in the first generation and phenotype CRISPR‐mediated edited plants before reproduction (exceptions are also found in Van Zeijl et al., 2018 and Varkonyi‐Gasic et al., 2019). Intriguingly, annualization of woody perennials has been reported recently in kiwifruit (*Actinidia chinensis* Planch.) by CRISPR‐mediated genome editing of *CEN*‐like genes (Varkonyi‐Gasic et al., 2019). Generation of early flowering kiwifruit accelerates its breeding and shows an excellent example of how CRISPR technology could facilitate potential future genetic studies in woody perennials (Varkonyi‐Gasic et al., 2019). In addition, applying a CRISPR system in a species with a short juvenile period would provide unprecedented potential for genetic research in its phylogenetically closely related woody perennials. Zhu et al. (2019) demonstrated CRISPR‐mediated genome editing in early flowering Hong Kong kumquat (*Citrus japonica* Thunb.; Rutaceae); both T_0_ and T_1_ genome‐edited plants were generated. Compared to a juvenile period of 5–10 years in other citrus species, Hong Kong kumquat blossomed eight months after seed sowing and could be used as a model species for citrus research (Zhu et al., 2019).

## TARGET GENE SELECTION

Target genes are selected in nongenetic model plant systems on a case‐by‐case basis, depending on the biological question(s) to be answered as well as characters of potential target genes (e.g., the copy number within the genome and phenotyping efficiency of the mutant, as described above). To identify the target gene sequences and predict the intron‐exon structure in nongenetic models, transcriptomic data can be used for BLAST searches against homologs from genetic model plant species; PCR amplification of corresponding genomic regions and sequencing the amplicons provide additional information for sgRNA design (Iaffaldano et al., 2016; Shan et al., 2018).

Generating a loss‐of‐function mutant by introducing a DSB at the exonic region of the target gene is the most common, efficient, and straightforward CRISPR application (reviewed in Zhang et al., 2017; Modrzejewski et al., 2019) and can be implemented in initial proof‐of‐principle studies of CRISPR technology in nongenetic models. For example, many CRISPR studies have focused on agronomic and/or economically beneficial traits, which are usually controlled by negative regulatory genes with one copy in the genome. Therefore, improved traits will be obtained through simple gene knockouts (see above).

In addition, knocking out a marker gene (i.e., a gene for which the mutant has an obvious phenotype and which allows easy visual screening) has been implemented as the first application of CRISPR technology in many plant species. Among various marker genes, the phytoene desaturase gene (*PDS*) is most popular (reviewed in Zhang et al., 2017). *PDS* encodes an essential enzyme that participates in the carotenoid biosynthesis pathway, and the loss‐of‐function mutant of *PDS* has an albino phenotype (Qin et al., 2007). In addition, *PDS* is typically a single‐copy gene in plant genomes, which is another advantage in its use (Shan et al., 2018). For these reasons, a *pds* mutant can be efficiently generated and visually identified.

Furthermore, the genome editing ability of CRISPR/Cas9 can be evaluated by mutagenesis of a transgene (Kishi‐Kaboshi et al., 2017). Kishi‐Kaboshi et al. (2017) designed sgRNAs to target the *yellowish‐green fluorescent protein* gene from the marine copepod *Chiridius poppei* (*CpYGFP*) in transgenic *Chrysanthemum* L. Given the availability of the *CpYGFP* sequence, the spacer sequence of sgRNA can be easily designed. The absence of fluorescence signals indicated that the *CpYGFP* has been inactivated by CRISPR in transgenic *Chrysanthemum* (Kishi‐Kaboshi et al., 2017).

## VECTOR CONSTRUCTION

The components of the CRISPR vector applied in a nongenetic model plant system could be adapted from a phylogenetically closely related genetic model species. Useful CRISPR plasmid information is available on Addgene (https://www.addgene.org).

### sgRNA

The sgRNA spacer sequence is complementary to the CRISPR/Cas9 target site, which is a prerequisite for introducing a DSB by the Cas9 endonuclease (Fig. 1). In nongenetic model plant systems, a target site(s) can be found manually by following the guidelines below:

- The length of the spacer sequence of a sgRNA is 20 nucleotides in most cases. In addition, shorter (e.g., 18 nucleotides from Nishitani et al., 2016) and longer (e.g., 24 nucleotides from Svitashev et al., 2015) spacer sequences have also been successfully used for genome editing. Fu et al. (2014) showed that truncated sgRNA with shorter spacer sequences (17–18 nucleotides) decreased off‐target mutations.
- The Cas9‐sgRNA complex can bind to either the coding strand (5′‐CCN‐N(20)‐3′) or the template strand (5′‐N(20)‐NGG‐3′) of a gene.
- To knock out a target gene, the CRISPR/Cas9 target site is usually constrained to the first few exons that precede or are located within the genomic sequence(s) encoding the functional domain(s) of the gene product (Shan et al., 2014).
- The spacer sequence of a sgRNA should not contain polyT, which is the transcription termination signal for RNA polymerase III (Miao et al., 2013).
- At least two sgRNAs per target gene should be designed, in case certain sgRNAs fail to work (Shan et al., 2014).
- High GC content (50–70%) of the CRISPR/Cas9 target site enhances the interaction between sgRNA and the target DNA, which might favor targeting efficiency, but also might lead to a higher risk of off‐targeting (Jao et al., 2013; Xie et al., 2014; Ma et al., 2015; Tsai et al., 2015).
- The spacer sequence of a sgRNA should not pair with the sgRNA scaffold with more than six nucleotides (Ma et al., 2015). The formation of a stem‐loop structure within the sgRNA may impact its binding capacity with the CRISPR/Cas9 target site (Ma et al., 2015).
- A specific first nucleotide (A and G for *U3* and *U6* small nuclear RNA [snRNA] gene promoters, respectively) enhances expression and stability of the sgRNA (Li et al., 2013; Shan et al., 2013, 2014; Bortesi and Fischer, 2015). The specific nucleotide can be either appended to the 5′ end of the 20‐nucleotide spacer sequence (e.g., 5′‐G‐N(20)‐3′) or serve as the first nucleotide of the spacer sequence (5′‐G‐N(19)‐3′) (may or may not match the genomic sequence).
- If multiple sgRNAs are arranged in a single expression vector, loss and rearrangement of sgRNA components can be avoided by using different RNA polymerase III‐dependent promoters (e.g., rice U6.1p and U6.2p; Zhou et al., 2014).
- The targeting specificity is determined by the 10 nucleotides immediately upstream of PAM in the spacer sequence of a sgRNA; off‐target effects might occur in DNA regions with variation of a few nucleotides within the PAM‐distal region (Ma et al., 2016).

sgRNAs are usually driven by a *U6* or *U3* snRNA gene promoter (hereafter referred to as U6 and U3 promoter, respectively [see Box 1]; transcribed by RNA polymerase III) and followed mostly by a polyT (five to eight Ts) transcription termination signal (Fig. 2). As we summarize in Fig. 2 and Appendix S1, the U6 promoter from *Arabidopsis thaliana* (L.) Heynh. is most widely used in eudicots; in monocots, *Oryza* U3 and U6 promoters have been widely used. The activities of different sgRNA promoters have been compared. In *Arabidopsis*, the U6 promoter had a higher activity compared with the U3 promoter (Zhang et al., 2016b); similar patterns have been identified in *Oryza* (Mikami et al., 2015) and *Camelina* (Morineau et al., 2017). Although the *Arabidopsis* and *Oryza* U6 promoters are widely used in eudicots and monocots, respectively (Fig. 2), species‐customized endogenous U6 promoters have been used in several plant species, including *Cichorium* L., *Glycine* Willd., *Lotus* L., *Medicago* L., and *Physcomitrella* Bruch & Schimp. (Appendix S1). In several species, the endogenous U6 promoter led to a higher mutation efficiency compared with the *Arabidopsis* counterpart (Sun et al., 2015; Andersson et al., 2017).

In terms of the scaffold of the sgRNA, a single RNA chimera of 76 nucleotides has been used in most CRISPR studies in plants (Appendix S1). Compared with dual crRNA:tracrRNA (Box 1), sgRNA can be relatively easily designed (Jinek et al., 2012) and has shown a higher genome editing efficiency (Mali et al., 2013; Miao et al., 2013). In addition, the long version of the sgRNA scaffold (76 nucleotides) showed a higher efficiency than the short version (42 nucleotides) (Hsu et al., 2013; Zhou et al., 2014).

### Multiplex editing

Functionally redundant genes or gene families play a pivotal role in fine tuning cellular processes; many agronomically beneficial traits are quantitative and controlled by multiple genes. Therefore, the application of CRISPR technology for simultaneous manipulation of multiple genes (i.e., multiplex editing) is of significance in both basic and applied research. Multiple sgRNA‐expressing cassettes can be stacked in a single construct using either regular cloning, Golden Gate cloning, or Gibson assembly (reviewed in Ma et al., 2016). Alternatively, Xie et al. (2015) engineered an endogenous tRNA‐processing system to generate multiple sgRNAs in vivo from a single transcript. In the plasmid construct, multiple sgRNAs are separated by tRNAs. The primary transcript from the construct will be cleaved by the endogenous RNase at tRNA–sgRNA junctions to release sgRNAs in vivo. As the tRNA‐processing system is universal in all living organisms, this system has broad applications in multiplex genome editing (Xie et al., 2015).

### Cas9 endonuclease

Most studies have used a codon‐optimized *SpCas9* gene (Fig. 2, Appendix S1) (an exception is also found in Jiang et al., 2013). Among the 45 genera summarized in Fig. 2, *Arabidopsis* codon‐optimized *Cas9* has been widely used in eudicots. Many CRISPR studies used *Homo sapiens* codon‐optimized *Cas9*, which worked well in both monocots and eudicots (Fig. 2). Codon‐optimized *Cas9* genes from *Oryza* and *Zea* L. have been applied in both monocot and eudicot CRISPR studies, but mostly in monocots (Fig. 2).

Although codon‐optimized *Cas9* genes for *Arabidopsis*,* Homo*, and *Oryza* have been widely used, a species‐specific codon‐optimized *Cas9* gene has been shown to lead to higher *Cas9* expression and can be designed for a nongenetic model plant species. In *Arabidopsis*, use of the *Arabidopsis* codon‐optimized *Cas9* showed a higher expression level compared to the human codon‐optimized *Cas9* (Li et al., 2013). In rice, the rice codon‐optimized *Cas9* gene showed a higher genome editing efficiency compared to the original bacterial *Cas9* gene, the human codon‐optimized *Cas9*, and the *Chlamydomonas* codon‐optimized *Cas9* gene (Zhou et al., 2014). Similarly, in *Zea mays* L., maize codon‐optimized *Cas9* performed better than the human codon‐optimized *Cas9* (Xing et al., 2014). To design a codon‐optimized *Cas9* gene, GenScript (Piscataway, New Jersey, USA) provides codon usage frequency tables for some widely studied plant species (https://www.genscript.com/tools/codon-frequency-table). In addition, several programs (e.g., CodonW [http://sourceforge.net/projects/codonw]) are available to assess the preferentially used codons in any organism of interest.

Constitutive and strong promoters are most widely used to drive *Cas9* gene expression. Based on our survey of CRISPR systems in 45 plant genera (Fig. 2, Appendix S1), *Cauliflower mosaic virus 35S* promoter (CaMV 35S) is the most commonly used promoter and has been widely applied in CRISPR studies of both monocots and eudicots. In addition, *ubiquitin* promoters have been broadly used to drive the *Cas9* gene, and in many studies, *ubiquitin* promoters exhibit higher genome editing efficiency than CaMV 35S (Ma et al., 2016). Specifically, the *Petroselinum crispum ubiquitin* promoter is widely used in eudicots, for instance, in *Arabidopsis*,* Lactuca* L., and *Vitis*. In monocots, the *Zea mays ubiquitin* promoter has been applied in the CRISPR systems of *Oryza*,* Panicum* L., *Triticum* L., and *Zea*. Furthermore, tissue‐ or cell‐specific promoters have been utilized to drive *Cas9* expression to increase the mutation efficiency and diversity, including meristem‐specific *Yao* promoter (Yan et al., 2015), egg cell‐specific *EC1.2* promoter (Wang et al., 2015), and germline‐specific *DD45* and *LAT52* promoters (Mao et al., 2016).

In terms of the terminator of *Cas9*, the nopaline synthase (*NOS*) gene terminator has been most widely used in both monocots and eudicots, and the *Pisum sativum* pea3A terminator has been broadly applied in CRISPR studies of eudicots (Fig. 2, Appendix S1). In addition, nuclear localization signals (NLS) are attached to a Cas9 protein to ensure the proper transport of the Cas9 endonuclease to the nucleus (Fig. 2; Li et al., 2013; Shan et al., 2013). A FLAG (Box 1) tag is usually fused with Cas9, and consequently the presence of Cas9 in transfected cells or transformed plants can be examined by an immunoblot analysis with an anti‐FLAG antibody (Li et al., 2013; Upadhyay et al., 2013; Xie and Yang, 2013). Furthermore, the *Cas9* gene can be engineered to incorporate an intron segment, which can minimize the negative effects of Cas9 in *Escherichia coli* and *Agrobacterium* cells (which do not incorporate the RNA splicing process during transcription) during vector construction (Jiang et al., 2013; Li et al., 2013; Svitashev et al., 2015). Lastly, compared with the separate delivery of sgRNA and *Cas9* into plant cells, co‐delivery of the two CRISPR components (the sgRNA and the *Cas9* gene are subcloned into a single construct; Fig. 2) has shown improved genome editing efficiency (Upadhyay et al., 2013).

## TRANSIENT ASSAY

### Approaches of transient assay

**Figure 3:**
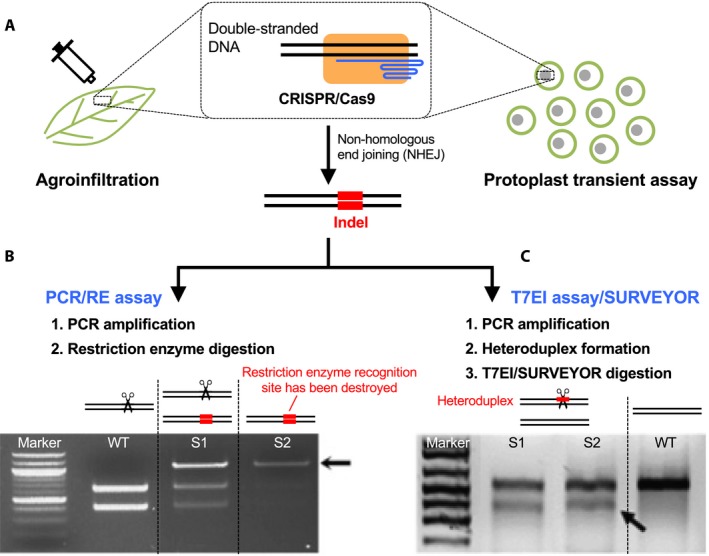
Transient assays for demonstrating the genome editing ability of CRISPR and strategies of identifying CRISPR‐induced mutations. (A) Agroinfiltration and protoplast transient assay are the two most widely used approaches of proof‐of‐principle CRISPR studies for identifying a desired CRISPR/Cas9 system for a new plant system of interest. The CRISPR‐induced indel at the target region can be detected either through PCR/RE assay or T7EI assay. (B) PCR/RE assay leverages the presence of a restriction enzyme target site within the CRISPR/Cas9 target site. After PCR amplification, wild type alleles will be cleaved (represented by the scissors) in the presence of the restriction enzyme. However, the enzyme cannot digest the mutated alleles in which the restriction enzyme recognition site has been destroyed by the NHEJ pathway. Therefore, the presence of the uncleaved band demonstrates the genome editing ability of CRISPR. The photograph of gel electrophoresis is modified from Fig. S6 in Zhang et al. (2019b) with permission; the arrow indicates the uncleaved PCR product, and S1 and S2 represent transgenic lines. (C) T7EI (or SURVEYOR) endonuclease is an enzyme sensitive to the mismatch sequences of double‐stranded DNA. If the CRISPR system works, indels will be introduced at the CRISPR target site. PCR amplicons are denatured and then re‐annealed. The heteroduplex containing unpaired nucleotides will be cleaved by T7EI/SURVEYOR. The photograph of gel electrophoresis is modified from Fig. 2 in Zhou et al. (2014) with permission; the arrow indicates the expected T7E1 cleavage product, and S1 and S2 represent transgenic lines. WT = wild type.

We recommend using transient assays to identify a desired CRISPR system for a newly studied plant species before the implementation of stable transformation. Compared with the laborious and time‐consuming (usually several months) process of plant transformation, transient assays are more convenient, and the results can be obtained in just a few days. A suitable CRISPR system can be developed de novo (e.g., Zhou et al., 2014) or adapted from a CRISPR system that has been developed in an evolutionarily closely related species (e.g., Shan et al., 2018). As discussed above, different CRISPR systems vary in the species‐specific codon‐optimized *Cas9* gene, and promoters and terminators of the *Cas9* and sgRNA genes. Two main approaches of transient assays, leaf cell agroinfiltration (e.g., Jiang et al., 2013; Li et al., 2013; Nekrasov et al., 2013; Upadhyay et al., 2013; Jia et al., 2017) and protoplast transfection (e.g., Feng et al., 2013; Jiang et al., 2013; Li et al., 2013; Xie and Yang, 2013; Liang et al., 2014; Andersson et al., 2017; Liu et al., 2018; Shan et al., 2018), have been widely used in developing new CRISPR systems (Fig. 3A). Moreover, in some species, transfected protoplasts can then be used for plant regeneration (Woo et al., 2015; Andersson et al., 2017; Collonnier et al., 2017; Osakabe et al., 2018). In addition, *Agrobacterium rhizogenes–*mediated hairy root transformation has been adopted for rapid assessment of a CRISPR system's efficiency in legume species, as well as *Cichorium*,* Salvia* L., and *Taraxacum* F. H. Wigg. (Jacobs et al., 2015; Iaffaldano et al., 2016; Wang et al., 2016; Li et al., 2017a; Bernard et al., 2019; Yuan et al., 2019).

The procedures of agroinfiltration and protoplast transient assays are briefly described below. For agroinfiltration, suspensions of *Agrobacterium* containing a plasmid expressing the *Cas9* and sgRNA genes can be introduced into plant leaves through either direct injection or vacuum infiltration. Subsequently, *Agrobacterium* cells transfer their transfer DNAs (T‐DNAs), which contain the CRISPR components, into the host plant cells. Genome editing events may then take place in transformed leaf cells. Protocols for efficient and routine agroinfiltration have been developed in several plant species, including representatives of *Arabidopsis*,* Lactuca*,* Nicotiana*, and *Solanum* L. (Wroblewski et al., 2005). Usually, the T‐DNA segment contains a reporter gene (e.g., the *GFP* gene; Jiang et al., 2013; Jia et al., 2017), and therefore, transformed cells can be readily identified. The process of protoplast transient assay includes protoplast isolation, transfection of plasmid DNA, and protoplast culture (Yoo et al., 2007; Zhang et al., 2011; Lin et al., 2018). Cellulase and macerozyme are commonly used to digest plant cell walls and isolate protoplasts. Plasmids containing CRISPR components are taken up by protoplasts through various methods, including polyethylene glycol (PEG)–calcium fusion, electroporation, and microinjection. The proper expression of the CRISPR components in transfected protoplasts will potentially introduce targeted genome editing in the genomic DNA.

### Endogenous gene editing

Transient assays have been mostly used to knock out an endogenous gene within a plant genome, which can be readily identified by either PCR/restriction enzyme (PCR/RE) assay (Jiang et al., 2013; Liang et al., 2014; Shan et al., 2014; also known as RFLP analysis in Feng et al., 2013 and Woo et al., 2015) or T7E1 assay (Xie and Yang, 2013; Shan et al., 2014) (Fig. 3). These two methods can be used to examine targeted mutations in stable transformants as well (Shan et al., 2014). The PCR/RE assay leverages the presence of a restriction enzyme recognition site at the Cas9 cutting site (three nucleotides upstream of PAM). The genomic DNA of transformed leaf cells (e.g., Li et al., 2013) or transfected protoplasts (e.g., Shan et al., 2013) will be isolated and PCR‐amplified using primers flanking the CRISPR/Cas9 target region (300–600‐bp amplicon as suggested by Shan et al., 2014). The amplicons will then be digested by the restriction enzyme that recognizes the restriction site. Amplicons from wild type alleles will be digested, and amplicons from the mutated alleles, in which the enzyme recognition sites have been destroyed by CRISPR‐induced mutations, will remain uncleaved (Fig. 3B). The intensity of the undigested DNA band indicates the genome editing efficiency of CRISPR (method in Shan et al., 2014). The mutated alleles can be further characterized by subcloning and sequencing the undigested amplicons. Alternatively, if the CRISPR/Cas9‐induced mutation frequency is relatively low, the mutated alleles can be enriched first by restriction digestion of the genomic DNA from transformed cells before the PCR/RE assay (Nekrasov et al., 2013; Shan et al., 2014; Lawrenson et al., 2015). In this case, mutated alleles without the restriction enzyme recognition site are preferentially amplified.

If there is no suitable restriction enzyme recognition site within the CRISPR/Cas9 target region, the T7EI system can be used to detect genome editing events introduced by CRISPR (Fig. 3C). T7EI nuclease (as well as SURVEYOR nuclease) is an enzyme that is sensitive to the mismatch sequences of double‐stranded DNA (Mao et al., 2013; Shan et al., 2014). PCR products (usually 300–600 bp) amplified by primers flanking the CRISPR target region are denatured and re‐annealed. Re‐annealed double‐stranded DNA containing unpaired nucleotides (i.e., heteroduplex) will be digested by T7EI or SURVEYOR nuclease (Fig. 3C). The more efficient the CRISPR system, the greater prevalence of the mutated alleles, and therefore the more re‐annealed PCR products will be digested. In addition, different mutation types can be further determined by subcloning and sequencing. Lastly, if the mutation rate is high in the transformants, without PCR/RE and T7EI assays, the amplicons covering the CRISPR/Cas9 target site can be randomly cloned and sequenced. The ratio of mutated clones to all sequenced clones can be used to infer the mutation efficiency of the CRISPR system.

### Exogenous gene editing

**Figure 4:**
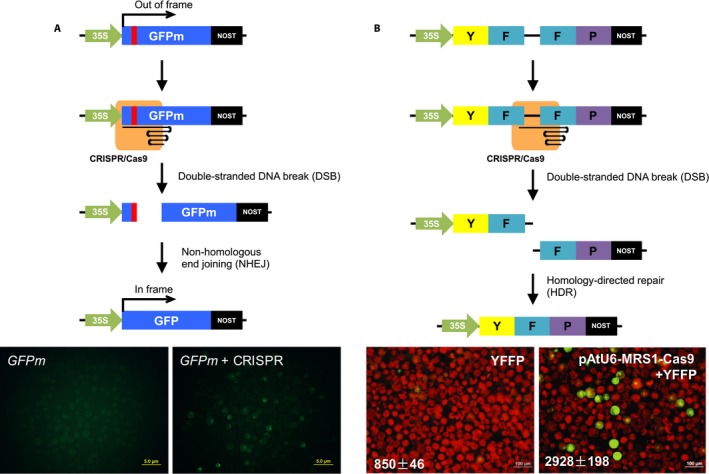
Schematic diagrams of CRISPR/Cas9‐mediated mutagenesis of exogenous reporter genes. (A) With an extra nucleotide (shaded in red) insertion upstream of a PAM sequence, the frameshift mutant *GFP* gene (*GFPm*) does not produce fluorescence signal. A DSB will be introduced by CRISPR around the position of the extra nucleotide in *GFPm*. The DSB is repaired by the NHEJ pathway, and indels will be introduced at the target site. Some indels, for instance a 1‐bp deletion and a 4‐bp deletion, will restore the reading frame of a functional *GFP* gene. Protoplast transient assay images are from *Tragopogon miscellus* under fluorescent light. The plasmid(s) used for transfection are shown at the top. (B) Partially overlapped yellow fluorescent protein (YFP) fragments are separated by a known linkage DNA, which contains a CRISPR/Cas9 target site. Guided by the sgRNA sequence, Cas9 introduces a DSB in the linkage DNA. Because of the sequence similarity between the two *YFP* fragments, a functional *YFP* gene can be generated through the HDR pathway. Images of the protoplast transient assay are modified from Fig. 6 in Zhang et al. (2016b) with permission. The plasmid(s) used for transfection are shown at the top; the average fluorescence intensity ± standard deviation is shown at the bottom. 35S represents *Cauliflower mosaic virus 35S* promoter; NOST indicates termination sequence of the nopaline synthase gene.

In addition to endogenous gene editing, an exogenous gene can be introduced into plant cells as a target of CRISPR in transient assays (Fig. 4). Because the sequence of the exogenous gene is already known, the advantage of this approach lies in the facile design of sgRNA. The exogenous gene is usually a mutated reporter gene, and therefore the function restoration of the reporter gene can be detected efficiently, which indicates the presence of genome editing events mediated by CRISPR. Frequently used exogenous genes include: frameshift mutant green fluorescence protein gene (*GFPm*) (Jiang et al., 2013; Liu et al., 2018; Shan et al., 2018), frameshift mutant red fluorescence protein (*DsRED2*) (Jiang et al., 2013), split yellow fluorescence protein (*YFFP*) (Feng et al., 2013; Mao et al., 2013), and split β‐glucuronidase (*GUUS*) (Mao et al., 2013; Miao et al., 2013). In terms of frameshift mutant reporter genes (e.g., *GFPm* and *DsRED2*), if a DSB is introduced by CRISPR and repaired by the NHEJ pathway, the function of the out‐of‐frame reporter gene might be restored, and readouts of CRISPR‐mediated editing events can be readily identified (Fig. 4A). In contrast, the YFFP and GUUS reporter systems utilize the HDR pathway to restore the function of the *YFP* and *GUS* reporter gene, respectively (Fig. 4B). In addition to transient assays, these reporter systems can also be applied to studies of stable transformation (Jiang et al., 2013; Mao et al., 2013; Miao et al., 2013).

## PLANT TRANSFORMATION

Plant genetic transformation is the process of introducing and expressing foreign gene(s) (i.e., *Cas9* and sgRNA in CRISPR studies) in plants (Christou, 1996; Birch, 1997). Here, we are focusing on stable transformation, in a process in which the foreign DNA is integrated into the plant genome and a transgenic plant is regenerated. The processes of plant transformation comprise: (1) identify explants with regeneration ability; (2) develop an efficient system to introduce foreign DNA into the explant cells; and (3) select successful transformants and regenerate plants from the transformants.

**Figure 5:**
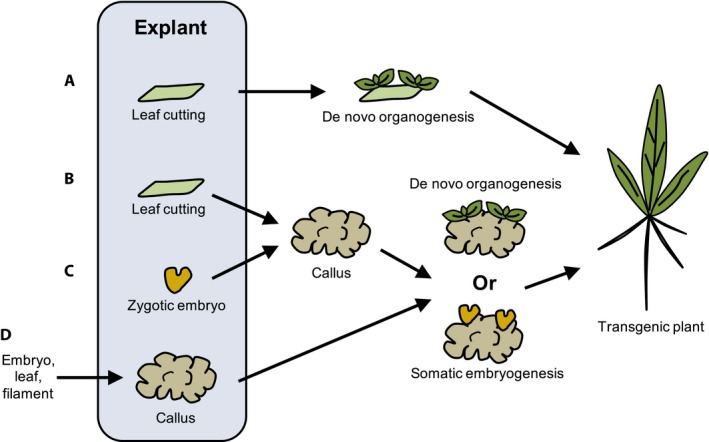
Different plant transformation strategies applied in CRISPR/Cas9 studies. Strategy (A) has been applied in regenerating CRISPR‐mediated transgenic plants in *Glycine* (Bao et al., 2019), *Nicotiana* (Jansing et al., 2019), *Solanum tuberosum* (Butler et al., 2015), *S. lycopersicum* (Nekrasov et al., 2017), *Cucumis sativus* (Chandrasekaran et al., 2016), and *Citrullus lanatus* (Tian et al., 2017). Strategy (B) has been used in studies of *Tragopogon* (Shan et al., 2018), *Lactuca* (Bertier et al., 2018), *Medicago* (Čermák et al., 2017), *Brassica* (Lawrenson et al., 2015), *S. lycopersicum* (Ito et al., 2015), *Populus* (Fan et al., 2015), *Petunia* (Zhang et al., 2016a), and *Torenia* (Nishihara et al., 2018). Strategy (C) has been used in CRISPR/Cas9 studies of *Triticum* (Zhang et al., 2019b), *Sorghum* (Liu et al., 2019), *Hordeum* (Lawrenson et al., 2015), *Zea* (Svitashev et al., 2015), and *Lotus* (Wang et al., 2016). Strategy (D) has been applied in *Oryza* (Zhou et al., 2014), *Panicum* (Liu et al., 2018), *Vitis* (Nakajima et al., 2017), *Manihot* (Odipio et al., 2017), and *Gossypium* (Gao et al., 2017).

Plant transformation is a prerequisite and, in most cases, a bottleneck for developing genome editing technology in any plant system (Altpeter et al., 2016). Transformation is also a major hurdle for applying a CRISPR system in nongenetic models. However, the procedure of plant transformation used in a phylogenetically closely related species may serve as a reliable reference and a reasonable starting point for developing a transformation system in a new plant system of interest (Birch, 1997). Here, we summarize different plant transformation strategies used in recent CRISPR/Cas9 studies (Fig. 5); the methods used in genetic models might work well in closely related nongenetic models.

### Explant selection and regeneration

An explant is a part of a plant or tissue used for transformation experiments (Christou, 1996). Based on our summary, the most commonly used explants include calli (unorganized cell mass), leaf cuttings, and zygotic embryos (Fig. 5). Leaf cuttings have been used as explants in eudicot plant transformations, and calli have been used in both monocots and eudicots (Fig. 5). In monocots, zygotic embryos have most commonly been used as explants because somatic tissues are usually less regenerative (Fig. 5; Ikeuchi et al., 2016). Following transformation, the explant will regenerate a whole transgenic plant through either de novo organogenesis or somatic embryogenesis (Fig. 5; Brown and Thorpe, 1986; Duclercq et al., 2011; Ikeuchi et al., 2016; Loyola‐Vargas and Ochoa‐Alejo, 2018). In practical applications, de novo organogenesis is more often used than somatic embryogenesis (Duclercq et al., 2011). In addition, explants from juvenile plants have a high regenerative capacity (Ikeuchi et al., 2016).

During the in vitro process of plant regeneration, plant hormones (i.e., auxin and cytokinin) regulate the growth status of plant explants. In principle, low, medium, and high auxin to cytokinin ratios promote shoot regeneration, callus induction, and root regeneration, respectively (Skoog and Miller, 1957; Ikeuchi et al., 2013, 2016). Furthermore, 2,4‐dichlorophenoxyacetic acid (2,4‐D) has been utilized in embryogenic callus formation (Ikeuchi et al., 2016, 2019). The regeneration ability of explants also depends on genetic variation within a species, the age of the plant from which the explants are derived, and environmental conditions, including gelling agent, pH, nutrient composition, light, and temperature (Ikeuchi et al., 2016).

Other than the conventional plant transformation strategy, CRISPR/Cas9‐mediated transgenic plants can also be obtained through other methods (see below), although to date the generality of these methods beyond a small group of species is not clear. Floral‐dip methods have been successfully implemented in *Arabidopsis*,* Camelina*, and *Thlaspi* L. (all from Brassicaceae) to generate genome‐edited plants using CRISPR (Feng et al., 2013; Morineau et al., 2017; McGinn et al., 2019). Protoplasts can also be used as explants for stable plant transformation (Woo et al., 2015; Andersson et al., 2017; Collonnier et al., 2017; Osakabe et al., 2018). In addition, hairy roots are an excellent transformation model system for species of Fabaceae; this transient assay can be adopted to rapidly test the genome editing ability of a CRISPR system (see Transient Assay, above). Finally, non‐vascular land plants possess unique transformation systems, and CRISPR/Cas9‐mediated genome‐edited plants have been obtained in *Marchantia* L. (liverworts; Sugano et al., 2014) and *Physcomitrella* (mosses; Collonnier et al., 2017).

### CRISPR component delivery

There are two main approaches to deliver the CRISPR components into the regenerable explant cells: *Agrobacterium* infection (e.g., Feng et al., 2013; Zhou et al., 2014; Shan et al., 2018) and particle bombardment (e.g., Miao et al., 2013; Shan et al., 2013, 2014; Svitashev et al., 2015; Liu et al., 2019). The majority of CRISPR studies use *Agrobacterium*‐mediated transformation to obtain transgenic plants. Each delivery system has both advantages and disadvantages. The *Agrobacterium*‐mediated transformation system is relatively simpler to operate and will integrate a lower copy number of transgenes into a plant genome compared to particle bombardment (Birch, 1997; Svitashev et al., 2015). However, *Agrobacterium* itself may cause negative effects on plant tissues, including browning and necrosis (Altpeter et al., 2016). Compared to *Agrobacterium*‐mediated transformation, particle bombardment has been shown to be applicable in a wider range of genotypes within a species (Altpeter et al., 2016). However, the particle bombardment approach is expensive, which limits its availability. In addition, virus‐mediated transformation has also been utilized to deliver sgRNA(s) into transgenic Cas9‐expression plants (Ali et al., 2015; Yin et al., 2015). For example, in Yin et al. (2015), sgRNAs were expressed on a modified cabbage leaf curl virus vector; genome editing has been identified in both inoculated and non‐inoculated leaves.

Different formats of Cas9 can be manipulated during plant transformation, with Cas9 delivery as DNA, mRNA, or protein (Glass et al., 2018). Delivering Cas9 into plant cells in a DNA format is inexpensive, and the DNA integration is relatively stable. However, because of the sustained expression of *Cas9*, both on‐target and off‐target genome editing rates of this approach are higher than with the other two methods. Delivered in the format of mRNA, the Cas9 protein can be synthesized rapidly, but this approach is less stable as the mRNA might be easily degraded. Lastly, premixed Cas9 protein and sgRNA, namely ribonucleoproteins (RNPs), can be introduced into plant cells directly (Woo et al., 2015). This approach leads to the most immediate onset of genome editing events. However, the large size of Cas9 endonuclease makes the introduction process challenging, and the direct introduction of bacterial proteins into a eukaryotic cell may trigger immunological responses (Glass et al., 2018). Importantly, both the mRNA and protein formats do not introduce transgenes into the plant genome, which represents a huge advantage over the DNA delivery system, especially in agricultural applications.

### Identification of transformants

To select transformants containing CRISPR cassettes, the Cas9/sgRNA construct is co‐transferred into a plant genome with a selection marker gene (e.g., genes for resistance to hygromycin, kanamycin, or spectinomycin) and sometimes a reporter gene (e.g., the *GFP* gene; Jiang et al., 2013; Nekrasov et al., 2013; Shan et al., 2018). Any stable transformants obtained from the selection media should contain the Cas9/sgRNA construct; if a reporter gene (e.g., *GFP*) is co‐transferred into a plant genome along with the Cas9/sgRNA vector, successfully transformed cells will emit green fluorescence, which can be readily identified (Jiang et al., 2013; Nekrasov et al., 2013; Shan et al., 2018). When delivering Cas9/sgRNA in the form of DNA, to ensure the successful integration of the Cas9/sgRNA vector into the plant genome, PCR screening should be implemented. With the template genomic DNA from a stable transformant, a pair of primers can be used to amplify the CRISPR components; the presence of a band with the anticipated size of DNA indicates the integration of the Cas9/sgRNA construct into the plant genome (Zhou et al., 2014; Zhang et al., 2019b). In addition, if a FLAG tag is attached to Cas9, immunoblot assays with the anti‐FLAG antibody can be utilized to examine the presence of the Cas9 endonuclease in the transformants (Li et al., 2013; Upadhyay et al., 2013; Xie and Yang, 2013). All of the evidence above can be combined to select successful transformants that contain Cas9/sgRNA vectors.

## EVALUATION OF GENOME EDITING RESULTS

### Detection of CRISPR‐mediated mutations

CRISPR/Cas9‐mediated target gene editing can be determined at the DNA level indirectly and directly. In many studies, evidence from both methods is combined. Indirect approaches have been used to evaluate the genome editing results with high accuracy and throughput: the PCR/RE assay can be implemented to detect genome editing events if a restriction enzyme recognition site is next to the PAM sequence; if not, the T7E1 assay can be used to detect targeted mutation as only the heteroduplex containing unpaired nucleotides can be digested by the T7E1 endonuclease (see Transient Assay, above). In addition, PAGE electrophoresis can separate different CRISPR‐mediated mutated alleles at one‐base resolution (Li et al., 2017a). Similarly, Andersson et al. (2017) used capillary electrophoresis to distinguish PCR amplicons of different sizes (as small as 1 bp difference). However, both PAGE and capillary electrophoresis cannot detect nucleotide substitutions and fail to distinguish different mutated alleles of the same size (Andersson et al., 2017). In addition, if the loss‐of‐function mutants of the target gene (e.g., *PDS*,* AGO7*) show a distinctive phenotype, the genome editing events can be visually identified; the ratio of transgenic plants with the expected mutant phenotype to all transgenic plants represents the genome editing efficiency of CRISPR (Brooks et al., 2014; Zhang et al., 2016a).

**Figure 6:**
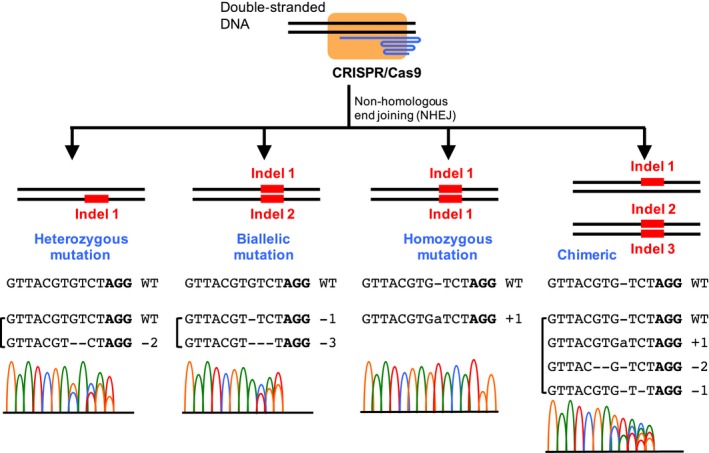
Various types of mutations mediated by CRISPR/Cas9. Each type is followed by the sequence(s) of mutated alleles (PAM sequence is in bold) and the corresponding sequencing chromatogram of the PCR product (different colors indicate different nucleotides). Multipeaks in the chromatogram are present in all mutation types except the homozygous mutation. Mutation features shown here include single‐base insertion (indicated as +1) and small deletions (such as −1 and −2), which are the most widely identified mutations mediated by CRISPR/Cas9. WT = wild type.

The specific features of mutations can only be revealed by direct sequencing. Both Sanger sequencing and high‐throughput sequencing of the amplicons derived from transformants/transgenic plants have been implemented to determine various mutation types, including heterozygous, biallelic, homozygous, and chimeric mutations (Fig. 6; reviewed in Ma et al., 2016). The features of mutations mediated by CRISPR/Cas9 are mostly single‐base insertions (mostly A and T) and small deletions (1–50 bp) (Fig. 6; Ma et al., 2016). Using Sanger sequencing, the PCR products can be either sequenced individually or as a whole if the genome editing is highly efficient. Individually, each allele can be examined by subcloning and Sanger sequencing. When the PCR products are sequenced together, the presence of multiple sequencing traces in sequencing chromatograms indicates the presence of multiple mutation types within the PCR products (Fig. 6). Although it is possible that not all allele types can be resolved by sequencing the amplicons together, the presence or absence of the wild type allele can be ambiguously determined (Shan et al., 2018). A program, DSDecode, has been developed to identify different allele types from a sequencing chromatogram file (Liu et al., 2015). In addition, high‐throughput sequencing has been applied in several studies for mutant identification, which facilitates identification of rare genome editing events and complicated mutations from chimeric individuals (Fauser et al., 2014; Svitashev et al., 2015; Ma et al., 2016; Zhang et al., 2019b).

### Detection of off‐target effects

The off‐target effect of CRISPR is challenging to predict and detect (Sander and Joung, 2014), but these effects have been found in a few plant species (Shan et al., 2013; Xie and Yang et al., 2013; Jacobs et al., 2015; Lawrenson et al., 2015). Off‐target mutations can be evaluated either at the whole‐genome level (e.g., Feng et al., 2014) or, more practically, within a few predicted potential off‐target locations (e.g., Zhou et al., 2014; Woo et al., 2015). In many cases, especially studies of *Arabidopsis* and crops, the off‐target effects of the CRISPR system have been comprehensively evaluated through deep sequencing of the entire genome (Feng et al., 2014; Sander and Joung, 2014; Tang et al., 2018). Feng et al. (2014) sequenced the complete nuclear genomes of CRISPR/Cas9 transgenic *Arabidopsis* plants, as well as their wild type counterparts. When the genomic sequences were mapped to the reference genome, the numbers of single‐nucleotide polymorphisms and indels did not differ between transgenic and wild type *Arabidopsis*, which implied that large‐scale off‐target mutations were absent in CRISPR/Cas9 transgenic *Arabidopsis* (Feng et al., 2014). If the high cost of whole‐genome sequencing is prohibitive, CRISPR‐mediated off‐target mutations can also be evaluated by selecting a few potential off‐target sites within the genome, a method that is efficient and affordable. Zhou et al. (2014) evaluated six potential off‐target sites; these sites included up to four mismatches to the CRISPR target region (20 bp in length) and were immediately upstream of the PAM sequence. Sanger sequencing of the amplicons from the six potential off‐target sites did not identify any off‐target events (Zhou et al., 2014).

### Inheritance of CRISPR‐mediated mutations

Evaluation of the heritability of CRISPR‐induced mutations or edits is of importance for both functional biology and crop improvement. In nongenetic models, stable inheritance of mutations allows phenotypic and genotypic examination of transgenic plants across multiple generations. However, not all mutations generated by CRISPR are heritable, as only editing in germline cells will be transmitted to the next generation. Mutations identified only in somatic cells, in the case of chimeric plants, will not be detected in subsequent generations (Feng et al., 2014; Jiang et al., 2014; Zhou et al., 2014; Morineau et al., 2017). In addition, *Agrobacterium*‐mediated transformation and particle bombardment incorporate vector sequences into the genomes of the initial generation of transgenic plants. To generate transgene‐free plants, backcrossing or selfing of the first‐generation transgenic plants is required, which, again, emphasizes the significance of evaluating the inheritance of CRISPR‐induced mutations.

The inheritance of CRISPR‐mediated mutations has been examined in many plant species (e.g., Feng et al., 2014; Jiang et al., 2014; Zhou et al., 2014; Braatz et al., 2017; Morineau et al., 2017; Bao et al., 2019; Zhang et al., 2019b). For example, in rice, as genome modification occurred in a single embryogenic cell, Zhou et al. (2014) showed that all mutations in T_0_ plants were heritable in the T_1_ progeny. The linkage of the CRISPR transgenes with the mutations introduced by CRISPR can be separated with the expected Mendelian inheritance in progenies following selfing (Zhou et al., 2014). The faithful inheritance of different mutation types between T_0_ plants and their progenies indicated that those genetic modifications adjacent to the PAM sequence prevent further editing of the target gene in subsequent generations (Zhou et al., 2014). In addition, multi‐copy genes may not all be inactivated by the CRISPR system at once within a single generation. Continued editing of the wild type allele demonstrates that the CRISPR system is functional in later generations, which enables both plant breeding and functional study of the target genes (Feng et al., 2014; Morineau et al., 2017; Bao et al., 2019).

## DISCUSSION

The development of CRISPR technology in nongenetic model plant systems will provide unprecedented insights into our understandings of biodiversity, adaptation, and evolution. In animals, CRISPR technology is creating a new wave of new model organisms and is also facilitating unique research opportunities, including studies of unusual camouflage and social behaviors (Reardon, 2019). Here we focus on applications in two groups of non‐model plants, *Tragopogon* and *Amborella*. *Tragopogon* belongs to Asteraceae (sunflower family) and represents a model for research on polyploidy; we discuss potential CRISPR applications to understand phenotypic diversity in Asteraceae and the genetic consequences of polyploidy. We then describe prospective CRISPR studies in the phylogenetically pivotal flowering plant *Amborella*, the sister to all other living angiosperms.

### Gene function and phenotypic diversity in Asteraceae

**Figure 7:**
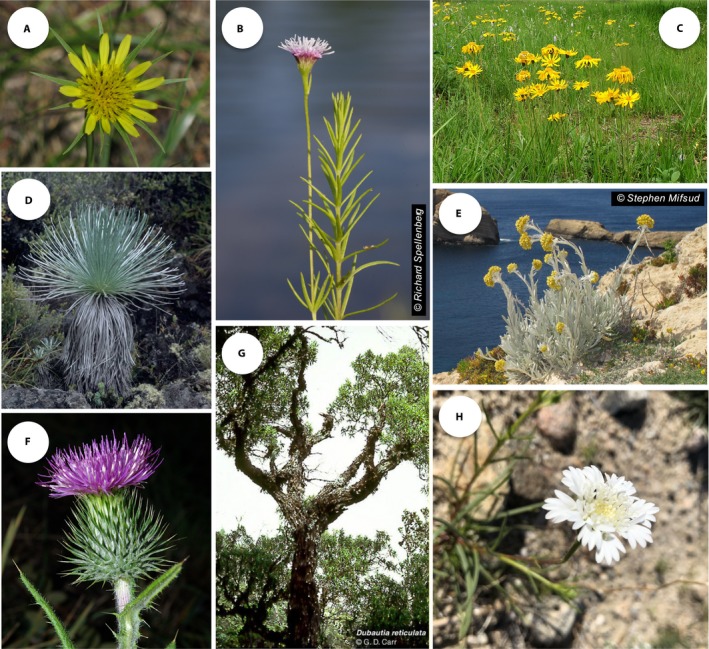
Morphological and habitat diversity of the sunflower family. (A) *Tragopogon dubius*; (B) *Sclerolepis uniflora*; (C) *Arnica montana*; (D) *Argyroxiphium kauense*; (E) *Helichrysum melitense*; (F) *Carduus acanthoides*; (G) *Dubautia reticulata*; (H) *Chaenactis fremontii*. Photo credits: (A) Jane S. Richardson (https://commons.wikimedia.org/wiki/File:Goats_beard_Tragopogon_dubius_close.jpg with modification); (B) with permission from Richard Spellenberg; (C) Bernd Haynold (https://commons.wikimedia.org/wiki/File:Arnica_montana_180605.jpg with modification); (D) Karl Magnacca (https://commons.wikimedia.org/wiki/File:Kahuku_silversword2.jpg with modification); (E) modified from Fig. 10A in Mifsud (2013) with permission; (F) David Perez (https://commons.wikimedia.org/wiki/File:Carduus_acanthoides_02_by-dpc.jpg with modification); (G) G. D. Carr (http://explorer.natureserve.org/servlet/NatureServe?searchName=Dubautia+reticulata); (H) David Rankin (https://www.inaturalist.org/photos/31291630 with modification).

Asteraceae are the largest or second largest family of flowering plants (~25,000 species; Judd et al., 2016) and include numerous crops, ornamentals, medicinal plants, and noxious weeds. The family is incredibly diverse morphologically, and species of the family occur in diverse habitats with highly variable underlying physiological adaptation (Fig. 7). In addition, the family is well known for its unique capitulum inflorescence.

These diverse features make Asteraceae an excellent choice for a diverse array of functional genetic studies by potential user groups representing both basic and applied research. For example, the functional genetics of inflorescence morphology could be explored in detail through analysis of candidate genes controlling features of floral and capitulum morphology. Exploring genes involved in floral symmetry could also be accomplished via a tractable CRISPR system for species of Asteraceae (e.g., *Tragopogon*). Morphological and physiological features associated with weediness could be explored in more detail, including plant vigor and rate of development, seed germination rates, seed size and dispersal, and drought tolerance. In addition, as the family is well known for its chemistry (Heywood et al., 1977), the genetic basis of the production of secondary metabolites (e.g., sesquiterpene lactones and latex) can be studied with CRISPR technology. Functional genetics of the unique sepals of the sunflower family, termed pappus, can be investigated with a tractable system and tools. For example, what genes function in the control of pappus morphology? Can the shape of the pappus be modified? Self‐incompatibility can also be explored in Asteraceae by using the CRISPR system: the genetic basis of self‐compatibility could be examined, and both self‐incompatible and self‐compatible forms of plants might be produced.

### Genetic consequences of polyploidy

Within *Tragopogon*, the availability of a workable CRISPR system will facilitate examination of gene function immediately following polyploidy (whole‐genome duplication [WGD]). For example, the gene balance hypothesis (e.g., Birchler and Veitia, 2007) suggests that following WGD, regulatory genes may be retained in duplicate to preserve dosage‐dependent stoichiometric relationships, while non‐regulatory genes may show patterns of fractionation (loss of one or the other parental homeolog). Which parental homeolog is ultimately retained may be determined by its connections to other genes in a network, although this hypothesis requires further study. But what would happen in terms of function and phenotype if one diploid parental gene copy were substituted for the other copy via CRISPR editing? Would this substitution disrupt normal gene function and development? Likewise, what is the effect of duplicate expression for a gene that expresses a single copy, or conversely, the effect of a single homeolog rather than duplicate expression?

An optimized CRISPR system would also provide an easy‐to‐use platform for researchers to address diverse questions of gene function and genotype–phenotype relationships following WGD. Polyploidy is often associated with an increase in the size of diverse plant traits, a result known as the gigas effect. For example, *Tragopogon* polyploids are much larger and more robust than their diploid parents; produce more inflorescences, flowers, and seeds; and also outcompete their parents (Novak et al., 1991). Polyploids are therefore excellent systems for the study of increased size, vigor, and morphological and physiological traits associated with the weedy habit. In addition to the above‐noted applications of CRISPR to studies of WGD, there are many other possible research opportunities, including subgenome dominance (Bird et al., 2018), biased fractionation, and cytonuclear interactions.

### Gene function and the origin of the flower: Applications in *Amborella*

The ABCE model (Box 1) of floral organ identity represents a major breakthrough of developmental genetics (Coen and Meyerowitz, 1991; Pelaz et al., 2000; Pinyopich et al., 2003; Ditta et al., 2004). The model explains how interacting patterns of gene expression control the formation of major floral organs—sepals, petals, stamens, and carpels. However, a strict ABCE model applies chiefly to eudicots. In many basal angiosperms and magnoliids, the floral organs are not well‐differentiated. For example, *Amborella*, the sister to all other living angiosperms, and many other non‐eudicots do not have distinct sepals and petals, but instead have tepals. The outermost floral organs are greenish and bract‐like, and these gradually transition to colorful petal‐like organs. Similarly, the stamens of *Amborella* and some other basal angiosperms and magnoliids are petal‐like. Rather than clearly differentiated patterns of gene expression in floral organs as found in *Arabidopsis* and most other eudicots, *Amborella* and other basal angiosperms show a gradual transition in gene expression across the floral meristem (e.g., Buzgo et al., 2004; Kim et al., 2005a, 2005b; Chanderbali et al., 2009). This pattern of overlapping expression of ABC genes has been referred to as the fading borders model (Buzgo et al., 2004; Soltis et al., 2007; Chanderbali et al., 2009).

If a CRISPR gene editing system were in place, the role of various floral organ identity genes could be rigorously examined in *Amborella*, as well as in other basal angiosperms (Nymphaeales and Austrobaileyales) and magnoliids. For example, what is the resultant floral morphology if critical floral organ identity genes such as *AP3*,* PI*, and *SEP* are individually knocked out? However, developing a de novo plant transformation system, including explant selection and regeneration, for *Amborella* as well as for many nongenetic models is challenging. In addition, as *Amborella* is a woody perennial, it will take several years for a transgenic line to reach maturity and flower, which would be another major hurdle for these studies (see Choice of Plant Species, above). Nevertheless, early flowering *Amborella* might be generated by targeted mutagenesis using CRISPR technology (such as mutagenesis of *CEN*‐like genes in *Actinidia*; Varkonyi‐Gasic et al., 2019). This approach might provide the plants needed to apply CRISPR to the study of flowering traits in this phylogenetically pivotal genus.

## AUTHOR CONTRIBUTIONS

All authors conceived the paper. S.S. drafted the outline. B.Y. and S.S. wrote the Introduction. D.E.S. and P.S.S. wrote the Discussion. S.S. wrote the remaining sections. All authors contributed to the final structure and editing of the manuscript.

## Supporting information


**APPENDIX S1.** References and construct information of 45 plant genera with established CRISPR systems.
