# Supplementary material for: Considerations in adapting CRISPR/Cas9 in nongenetic model plant systems
Source: Appl Plant Sci. 2020 Jan 12;8(1):e11314. doi: 10.1002/aps3.11314 (PMC6976890; doi:10.1002/aps3.11314)
Supplement: Supplementary file 1 — APPENDIX S1. References and construct information of 45 plant genera with established CRISPR systems. [file APS3-8-e11314-s001.docx]

**APPENDIX S1.** References and construct information of 45 plant genera with established CRISPR systems.

| **Genus (Family)** | **Reference** | **Cas9** | | | **sgRNA** | |
| --- | --- | --- | --- | --- | --- | --- |
|  |  | **Codon optimization** | **Promoter** | **Terminator** | **Scaffold** | **Promoter** |
| *Actinidia* (Actinidiaceae) | Varkonyi-Gasic et al., 2019 | *At* | *Pc ubiquitin* | *Ps* pea3A | 76 nt | *At* U3; *At* U6 |
| *Arabidopsis* (Brassicaceae) | Fauser et al., 2014 | *At* | *Pc ubiquitin* | *Ps* pea3A | 76 nt | *At* U6 |
|  | Feng et al., 2013 | *Hs* | CaMV *35S*^a^ | NOS^b^ | 76 nt | *At* U6 |
|  | Jiang et al., 2013 | *Cr* | CaMV *35S* | NOS | 76 nt | *At* U6 |
|  | Li et al., 2013 | *At* (most efficient^c^) | CaMV *35S* | NOS | 76 nt | *At* U6 |
|  | Mao et al., 2013 | *Hs* | *At* *ubiquitin* | *At ubiquitin* | 76 nt | *At* U6 |
| *Arachis* (Fabaceae) | Yuan et al., 2019 | *Hs* | CaMV *35S* | NOS | 76 nt | *Mt* U6 |
| *Brachypodium* (Poaceae) | Qin et al., 2019 | Plant-optimized codon (*Cas9p*)^d^ | *Zm* *ubiquitin* | NOS | NA | *Os* U3 |
| *Brassica* (Brassicaceae) | Braatz et al., 2017 | *At* | *Pc ubiquitin* | *Ps* pea3A | 76 nt | *At* U6 |
|  | Lawrenson et al., 2015 | *Hs* | CaMV *35S* | 35S^e^ | 76 nt | *At* U6 |
| *Camelina* (Brassicaceae) | Morineau et al., 2017 | *At* | *Pc ubiquitin* | *Ps* pea3A | 76 nt | *Cs* U3; *Cs* U6 |
| *Chrysanthemum* (Asteraceae) | Kishi-Kaboshi et al., 2017 | *At* | *Pc ubiquitin* | *Ps* pea3A | 76 nt | *At* U6 |
| *Cichorium* (Asteraceae) | Bernard et al., 2019 | Plant-optimized codon (*Cas9p*) | CaMV *35S* | 35S | 76 nt | *Ci* U6 |
| *Citrullus* (Cucurbitaceae) | Tian et al., 2017 | *Zm* | CaMV *35S* | NOS | 76 nt | *At* U6 |
| *Citrus* (Rutaceae) | Jia and Wang, 2014 | *Hs* | CaMV *35S* | NOS | 76 nt | CaMV *35S* |
|  | Jia et al., 2017 | *Hs* | CaMV *35S* | NOS | 76 nt | CaMV *35S* |
| *Coffea* (Rubiaceae) | Breitler et al., 2018 | *At* | CaMV *35S* | 35S | 76 nt | *Cc* U6 |
| *Cucumis* (Cucurbitaceae) | Chandrasekaran et al., 2016 | *At* | CaMV *35S* | NOS | NA | *At* U6 |
| *Daucus* (Apiaceae) | Klimek-Chodacka et al., 2018 | *At* (most efficient) | CaMV *35S* | NOS | NA | *At* U3 |
| *Fragaria* (Rosaceae) | Martín-Pizarro et al., 2018 | *Hs* | CaMV *35S* | NA | NA | *At* U6 |
| *Glycine* (Fabaceae) | Bao et al., 2019 | Plant-optimized codon (*Cas9p*) | CaMV *35S* | 35S | 76 nt | *At* U3; *At* U6 |
|  | Jacobs et al., 2015 | *Hs* | CaMV *35S* | NOS | 76 nt | *Mt* U6 |
|  | Li et al., 2015 | *Gm* | Soybean *EF1A2* | Potato proteinase inhibitor II | NA | *Gm U6* |
| *Gossypium* (Malvaceae) | Gao et al., 2017 | Plant-optimized codon (*Cas9p*) | CaMV *35S* | 35S | 76 nt | *At* U3; *At* U6 |
|  | Li et al., 2017b | *Zm* | CaMV *35S* | rbcS-E9 | 76 nt | *At* U6 |
| *Hordeum* (Poaceae) | Lawrenson et al., 2015 | *Hs* | *Zm* *ubiquitin* | NOS | 76 nt | ﻿*Ta* U6 |
| *Ipomoea* (Convolvulaceae) | Watanabe et al., 2017 | *At* | *Pc ubiquitin* | *Ps* pea3A | 76 nt | *At* U6 |
| *Lactuca* (Asteraceae) | Bertier et al., 2018 | *At* | *Pc ubiquitin* | *Ps* pea3A | 76 nt | *At* U6 |
| *Linum* (Linaceae) | Sauer et al., 2016 | Plant codon optimized Cas9 | Mannopine synthase promoter | rbcS-E9 | NA | *At* U6 |
| *Lotus* (Fabaceae) | Wang et al., 2016 | *Hs* | CaMV *35S* | NOS | 76 nt | *Lj* U6 |
| *Malus* (Rosaceae) | Nishitani et al., 2016 | Plant-optimized codon (*fcoCas9*)^f^ | CaMV *35S* | Heat shock protein 18.2 | NA | *At* U6 |
| *Manihot* (Euphorbiaceae) | Odipio et al., 2017 | None^g^ | CaMV *35S* | NOS | NA | *At* U6 |
| *Marchantia* (Marchantiaceae) | Sugano et al., 2014 | *Hs* | CaMV *35S* | NA | 76 nt | *Mp* U6 |
| *Medicago* (Fabaceae) | Gao et al., 2018 | Eudicot plants Cas9 | *Ubiquitin* | 35S | NA | At U6 |
|  | Meng et al., 2017 | *Os* | CaMV *35S* | NOS | 76 nt | *Mt* U6 |
| *Musa* (Musaceae) | Kaur et al., 2018 | *Hs* | CaMV *35S* | NOS | 76 nt | *Os* U3 |
| *Nicotiana* (Solanaceae) | Gao et al., 2015 | *Nt* | CaMV *35S* | NOS | 76 nt | *At* U6 |
|  | Jansing et al., 2019 | *At* | CaMV *35S* | NOS | NA | *At* U6 |
|  | Jiang et al., 2013 | *Cr* | CaMV *35S* | NOS | 76 nt | *At* U6 |
|  | Li et al., 2013 | *At* | CaMV *35S* | NOS | 76 nt | *At* U6 |
|  | Nekrasov et al., 2013 | *Hs* | CaMV *35S* | NA | 76 nt | *At* U6 |
|  | Upadhyay et al., 2013 | *Hs* | CaMV *35S* | NOS | 76 nt | CaMV *35S* |
|  |  |  |  |  |  |  |
| *Oryza* (Poaceae) | Feng et al., 2013 | *Hs* | CaMV *35S* | NOS | 76 nt | *Os* U6 |
|  | Jiang et al., 2013 | None; *Os* | CaMV *35S* | NOS | 42 nt | *Os* U6 |
|  | Mao et al., 2013 | *Hs* | *Os* *ubiquitin* | NOS | 76 nt | *Os* U3 |
|  | Miao et al., 2013 | *Os* | *Zm* *ubiquitin* | 35S | NA | *Os* U3 |
|  | Shan et al., 2013 | *Os* | CaMV *35S* | 35S | 76 nt | *Os* U3 |
|  | Xie and Yang, 2013 | *Hs* | RNA polymerase II | RNA polymerase II | 76 nt | *Os* U3; *Os* U6 |
|  | Zhou et al., 2014 | *Os* (most efficient) | *Zm* *ubiquitin* | NOS | 42 nt; 76 nt | *Os* U6 |
| *Panicum* (Poaceae) | Liu et al., 2018 | *Os* | *Zm* *ubiquitin* | NOS | 76 nt | *Os* U6 |
| *Papaver* (Papaveraceae) | Alagoz et al., 2016 | *Hs* | CaMV *35S* | NOS | 76 nt | *At* U6 |
| *Parasponia* (Cannabaceae) | Van Zeijl et al., 2018 | *At* | CaMV *35S* | 35S | 76 nt | *At* U6 |
| *Petunia* (Solanaceae) | Zhang et al., 2016 | *At* | CaMV *35S* | NOS | 76 nt | *At* U6 |
| *Physcomitrella* (Funariaceae) | Collonnier et al., 2017 | *Hs* | *Os* *Actin 1* | NOS | 76 nt | *Pp* U6 |
| *Populus* (Salicaceae) | Zhou et al., 2015 | *Hs* | CaMV *35S* | NOS | 76 nt | *Mt* U6 |
| *Salvia* (Lamiaceae) | Li et al., 2017a | *At* | CaMV *35S* | NA | 76 nt | *At* U6 |
| *Solanum* (Solanaceae) | Andersson et al., 2017 | *At* | CaMV *35S* | NOS | 76 nt | *St* U6 |
|  | Brooks et al., 2014 | *Hs* | CaMV *35S* | NOS | 76 nt | *At* U6 |
|  |  |  |  |  |  |  |
|  |  |  |  |  |  |  |
| *Sorghum* (Poaceae) | Jiang et al., 2013 | *Zm* | *Os* *Actin 1* | Octopine synthase gene | 76 nt | *Os* U6 |
|  | Liu et al., 2019 | NA | *Zm* *ubiquitin* | NOS | NA | *Os* U3 |
| *Taraxacum* (Asteraceae) | Iaffaldano et al., 2016 | Plant codon optimized Cas9 | CaMV *35S* | NOS | 76 nt | *At* U6 |
| *Theobroma* (Malvaceae) | Fister et al., 2018 | NA | CaMV *35S* | NOS | NA | *At* U6 |
| *Thlaspi* (Brassicaceae) | McGinn et al., 2019 | *At* | *Pc ubiquitin* | *Ps* pea3A | 76 nt | *At* U6 |
| *Torenia* (Linderniaceae) | Nishihara et al., 2018 | *At* | CaMV *35S* | Heat shock protein 18.2 | NA | *At* U6 |
| *Tragopogon* (Asteraceae) | Shan et al., 2018 | *At* | *At* *ubiquitin* | Octopine synthase gene | 76 nt | *At* U6 |
| *Triticum* (Poaceae) | Shan et al., 2013 | *Os* | CaMV *35S* | 35S | 76 nt | ﻿*Ta* U6 |
|  | Upadhyay et al., 2013 | *Hs* | CaMV *35S* | NOS | 76 nt | CaMV *35S* |
|  | Zhang et al., 2019 | *Ta* | *Zm* *ubiquitin* | NOS | 76 nt | *Ta* U6 |
| *Vitis* (Vitaceae) | Nakajima et al., 2017 | *At* | *Pc ubiquitin* | *Ps* pea3A | 76 nt | *At* U6 |
| *Zea* (Poaceae) | Liang et al., 2014 | *Os* | CaMV *35S* | 35S | 76 nt | *Zm* U3 |
|  | Svitashev et al., 2015 | *Zm* | *Zm* *ubiquitin* | Potato proteinase inhibitor II | 76 nt | *Zm* U6 |
|  | Xing et al., 2014 | *Zm* (most efficient) | CaMV *35S* | NOS | 76 nt | *Ta* U3 |

*Note:* NA = not available from the reference; nt = nucleotides; Species name abbreviations: *At = Arabidopsis thaliana*; *Cc* = *Coffea canephora*; *Ci* = *Cichorium intybus*; *Cr = Chlamydomonas reinhardtii*; *Cs = Camelina sativa*; *Gm = Glycine max*; *Hs = Homo sapiens*; *Lj = Lotus japonicus*; *Mp = Marchantia polymorpha*; *Mt = Medicago truncatula*; *Nt = Nicotiana tabacum*; *Os* = *Oryza sativa*; *Pc = Petroselinum crispum*; *Pp = Physcomitrella patens*; *Ps* = *Pisum sativum*; *St = Solanum tuberosum*; *Ta = Triticum aestivum*; *Zm* = *Zea mays*.

^a^*Cauliflower mosaic virus 35S* promoter.

^b^Termination sequence of the nopaline synthase gene.

^c^Multiple versions are tested and the most efficient version is listed in the table.

^d^*Cas9p* from Ma et al. (2015).

^e^35S terminator from *Cauliflower mosaic virus*.

^f^*fcoCas9* from Osakabe et al. (2016).

^g^The original *Cas9* sequence from *Streptococcus pyogenes* has been used*.*

**LITERATURE CITED**

Alagoz, Y., T. Gurkok, B. Zhang, and T. Unver. 2016. Manipulating the biosynthesis of bioactive compound alkaloids for next-generation metabolic engineering in opium poppy using CRISPR-Cas 9 genome editing technology. *Scientific Reports* 6: 30910.

Andersson, M., H. Turesson, A. Nicolia, A. S. Fält, M. Samuelsson, and P. Hofvander. 2017. Efficient targeted multiallelic mutagenesis in tetraploid potato (*Solanum tuberosum*) by transient CRISPR-Cas9 expression in protoplasts. *Plant Cell Reports* 36: 117-128.

Bao, A., H. Chen, L. Chen, S. Chen, Q. Hao, W. Guo, D. Qiu, et al. 2019. CRISPR/Cas9-mediated targeted mutagenesis of *GmSPL9* genes alters plant architecture in soybean. *BMC Plant Biology* 19: 131.

Bernard, G., D. Gagneul, H. Alves Dos Santos, A. Etienne, J. L. Hilbert, and C. Rambaud. 2019. Efficient genome editing using CRISPR/Cas9 technology in chicory. *International Journal of Molecular Sciences* 20: 1155.

Bertier, L. D., M. Ron, H. Huo, K. J. Bradford, A. B. Britt, and R. W. Michelmore. 2018. High-resolution analysis of the efficiency, heritability, and editing outcomes of CRISPR/Cas9-induced modifications of *NCED4* in lettuce (*Lactuca sativa*). *G3: Genes, Genomes, Genetics* 8: 1513-1521.

Braatz, J., H. J. Harloff, M. Mascher, N. Stein, A. Himmelbach, and C. Jung. 2017. CRISPR-Cas9 targeted mutagenesis leads to simultaneous modification of different homoeologous gene copies in polyploid oilseed rape (*Brassica napus*). *Plant Physiology* 174: 935-942.

Breitler, J. C., E. Dechamp, C. Campa, L. A. Z. Rodrigues, R. Guyot, P. Marraccini, and H. Etienne. 2018. CRISPR/Cas9-mediated efficient targeted mutagenesis has the potential to accelerate the domestication of *Coffea canephora*. *Plant Cell, Tissue and Organ Culture* 134: 383-394.

Brooks, C., V. Nekrasov, Z. B. Lippman, and J. Van Eck. 2014. Efficient gene editing in tomato in the first generation using the clustered regularly interspaced short palindromic repeats/CRISPR-associated9 system. *Plant Physiology* 166: 1292-1297.

Chandrasekaran, J., M. Brumin, D. Wolf, D. Leibman, C. Klap, M. Pearlsman, A. Sherman, et al. 2016. Development of broad virus resistance in non-transgenic cucumber using CRISPR/Cas9 technology. *Molecular Plant Pathology* 17: 1140-1153.

Collonnier, C., A. Epert, K. Mara, F. Maclot, A. Guyon-Debast, F. Charlot, C. White, et al. 2017. CRISPR-Cas9-mediated efficient directed mutagenesis and RAD51-dependent and RAD51-independent gene targeting in the moss *Physcomitrella patens*. *Plant Biotechnology Journal* 15: 122-131.

Fauser, F., S. Schiml, and H. Puchta. 2014. Both CRISPR/Cas-based nucleases and nickases can be used efficiently for genome engineering in *Arabidopsis thaliana*. *The Plant Journal* 79: 348-359.

Feng, Z., B. Zhang, W. Ding, X. Liu, D. L. Yang, P. Wei, F. Cao, et al. 2013. Efficient genome editing in plants using a CRISPR/Cas system. *Cell Research* 23: 1229-1232.

Fister, A. S., L. Landherr, S. N. Maximova, and M. J. Guiltinan. 2018. Transient expression of CRISPR/Cas9 machinery targeting *TcNPR3* enhances defense response in *Theobroma cacao*. *Frontiers in Plant Science* 9: 268.

Gao, J., G. Wang, S. Ma, X. Xie, X. Wu, X. Zhang, Y. Wu, et al. 2015. CRISPR/Cas9-mediated targeted mutagenesis in *Nicotiana tabacum*. *Plant Molecular Biology* 87: 99-110.

Gao, W., L. Long, X. Tian, F. Xu, J. Liu, P. K. Singh, J. R. Botella, and C. Song. 2017. Genome editing in cotton with the CRISPR/Cas9 system. *Frontiers in Plant Science* 8: 1364.

Gao, R., B. A. Feyissa, M. Croft, and A. Hannoufa. 2018. Gene editing by CRISPR/Cas9 in the obligatory outcrossing *Medicago sativa*. *Planta* 247: 1043-1050.

Iaffaldano, B., Y. Zhang, and K. Cornish. 2016. CRISPR/Cas9 genome editing of rubber producing dandelion *Taraxacum kok-saghyz* using *Agrobacterium rhizogenes* without selection. *Industrial Crops and Products* 89: 356-362.

Jacobs, T. B., P. R. LaFayette, R. J. Schmitz, and W. A. Parrott. 2015. Targeted genome modifications in soybean with CRISPR/Cas9. *BMC Biotechnology* 15: 16.

Jansing, J., M. Sack, S. M. Augustine, R. Fischer, and L. Bortesi. 2019. CRISPR/Cas9-mediated knockout of six glycosyltransferase genes in *Nicotiana benthamiana* for the production of recombinant proteins lacking β-1, 2-xylose and core α-1, 3-fucose. *Plant Biotechnology Journal* 17: 350-361.

Jia, H., and N. Wang. 2014. Targeted genome editing of sweet orange using Cas9/sgRNA. *PLoS ONE* 9: e93806.

Jia, H., Y. Zhang, V. Orbović, J. Xu, F. F. White, J. B. Jones, and N. Wang. 2017. Genome editing of the disease susceptibility gene *CsLOB1* in citrus confers resistance to citrus canker. *Plant Biotechnology Journal* 15: 817-823.

Jiang, W., H. Zhou, H. Bi, M. Fromm, B. Yang, and D. P. Weeks. 2013. Demonstration of CRISPR/Cas9/sgRNA-mediated targeted gene modification in *Arabidopsis*, tobacco, sorghum and rice. *Nucleic Acids Research* 41: e188.

Kaur, N., A. Alok, N. Kaur, P. Pandey, P. Awasthi, and S. Tiwari. 2018. CRISPR/Cas9-mediated efficient editing in *phytoene desaturase* (*PDS*) demonstrates precise manipulation in banana cv. Rasthali genome. *Functional & Integrative Genomics* 18: 89-99.

Kishi-Kaboshi, M., R. Aida, and K. Sasaki. 2017. Generation of gene-edited *Chrysanthemum morifolium* using multicopy transgenes as targets and markers. *Plant and Cell Physiology* 58: 216-226.

Klimek-Chodacka, M., T. Oleszkiewicz, L. G. Lowder, Y. Qi, and R. Baranski. 2018. Efficient CRISPR/Cas9-based genome editing in carrot cells. *Plant Cell Reports* 37: 575-586.

Lawrenson, T., O. Shorinola, N. Stacey, C. Li, L. Østergaard, N. Patron, C. Uauy, and W. Harwood. 2015. Induction of targeted, heritable mutations in barley and *Brassica oleracea* using RNA-guided Cas9 nuclease. *Genome Biology* 16: 258.

Li, J. F., J. E. Norville, J. Aach, M. McCormack, D. Zhang, J. Bush, G. M. Church, and J. Sheen. 2013. Multiplex and homologous recombination-mediated genome editing in *Arabidopsis* and *Nicotiana benthamiana* using guide RNA and Cas9. *Nature Biotechnology* 31: 688-691.

Li, Z., Z. B. Liu, A. Xing, B. P. Moon, J. P. Koellhoffer, L. Huang, R. T. Ward, et al. 2015. Cas9-guide RNA directed genome editing in soybean. *Plant Physiology* 169: 960-970.

Li, B., G. Cui, G. Shen, Z. Zhan, L. Huang, J. Chen, and X. Qi. 2017a. Targeted mutagenesis in the medicinal plant *Salvia miltiorrhiza*. *Scientific Reports* 7: 43320.

Li, C., T. Unver, and B. Zhang. 2017b. A high-efficiency CRISPR/Cas9 system for targeted mutagenesis in cotton (*Gossypium hirsutum* L.). *Scientific Reports* 7: 43902.

Liang, Z., K. Zhang, K. Chen, and C. Gao. 2014. Targeted mutagenesis in *Zea mays* using TALENs and the CRISPR/Cas system. *Journal of Genetics and Genomics* 41: 63-68.

Liu, Y., P. Merrick, Z. Zhang, C. Ji, B. Yang, and S. Z. Fei. 2018. Targeted mutagenesis in tetraploid switchgrass (*Panicum virgatum* L.) using CRISPR/Cas9. *Plant Biotechnology Journal* 16: 381-393.

Liu, G., J. Li, and I. D. Godwin. 2019. Genome editing by CRISPR/Cas9 in sorghum through biolistic bombardment. *In* Z. Y. Zhao and J. Dahlberg [eds.], Sorghum, 169-183. Humana Press, New York, New York, USA.

Ma, X., Q. Zhang, Q. Zhu, W. Liu, Y. Chen, R. Qiu, B. Wang, et al. 2015. A robust CRISPR/Cas9 system for convenient, high-efficiency multiplex genome editing in monocot and dicot plants. *Molecular Plant* 8: 1274-1284.

Mao, Y., H. Zhang, N. Xu, B. Zhang, F. Gou, and J. K. Zhu. 2013. Application of the CRISPR-Cas system for efficient genome engineering in plants. *Molecular Plant* 6: 2008-2011.

Martín-Pizarro, C., J. C. Triviño, and D. Posé. 2018. Functional analysis of the *TM6* MADS-box gene in the octoploid strawberry by CRISPR/Cas9-directed mutagenesis. *Journal of Experimental Botany* 70: 885-895.

McGinn, M., W. B. Phippen, R. Chopra, S. Bansal, B. A. Jarvis, M. E. Phippen, K. M. Dorn, et al. 2019. Molecular tools enabling pennycress (*Thlaspi arvense*) as a model plant and oilseed cash cover crop. *Plant Biotechnology Journal* 17: 776-788.

Meng, Y., Y. Hou, H. Wang, R. Ji, B. Liu, J. Wen, L. Niu, and H. Lin. 2017. Targeted mutagenesis by CRISPR/Cas9 system in the model legume *Medicago truncatula*. *Plant Cell Reports* 36: 371-374.

Miao, J., D. Guo, J. Zhang, Q. Huang, G. Qin, X. Zhang, J. Wan, et al. 2013. Targeted mutagenesis in rice using CRISPR-Cas system. *Cell Research* 23: 1233-1236.

Morineau, C., Y. Bellec, F. Tellier, L. Gissot, Z. Kelemen, F. Nogué, and J. D. Faure. 2017. Selective gene dosage by CRISPR-Cas9 genome editing in hexaploid *Camelina sativa*. *Plant Biotechnology Journal* 15: 729-739.

Nakajima, I., Y. Ban, A. Azuma, N. Onoue, T. Moriguchi, T. Yamamoto, S. Toki, and M. Endo. 2017. CRISPR/Cas9-mediated targeted mutagenesis in grape. *PLoS ONE* 12: e0177966.

Nekrasov, V., B. Staskawicz, D. Weigel, J. D. Jones, and S. Kamoun. 2013. Targeted mutagenesis in the model plant *Nicotiana benthamiana* using Cas9 RNA-guided endonuclease. *Nature Biotechnology* 31: 691-693.

Nishihara, M., A. Higuchi, A. Watanabe, and K. Tasaki. 2018. Application of the CRISPR/Cas9 system for modification of flower color in *Torenia fournieri*. *BMC Plant Biology* 18: 331.

Nishitani, C., N. Hirai, S. Komori, M. Wada, K. Okada, K. Osakabe, T. Yamamoto, and Y. Osakabe. 2016. Efficient genome editing in apple using a CRISPR/Cas9 system. *Scientific Reports* 6: 31481.

Odipio, J., T. Alicai, I. Ingelbrecht, D. A. Nusinow, R. Bart, and N. J. Taylor. 2017. Efficient CRISPR/Cas9 genome editing of *phytoene desaturase* in cassava. *Frontiers in Plant Science* 8: 1780.

Osakabe, Y., T. Watanabe, S. S. Sugano, R. Ueta, R. Ishihara, K. Shinozaki, and K. Osakabe. 2016. Optimization of CRISPR/Cas9 genome editing to modify abiotic stress responses in plants. *Scientific Reports* 6: 26685.

Qin, Z., Y. Bai, S. Muhammad, X. Wu, P. Deng, J. Wu, H. An, and L. Wu. 2019. Divergent roles of FT-like 9 in flowering transition under different day lengths in *Brachypodium distachyon*. *Nature Communications* 10: 812.

Sauer, N. J., J. Narváez-Vásquez, J. Mozoruk, R. B. Miller, Z. J. Warburg, M. J. Woodward, Y. A. Mihiret, et al. 2016. Oligonucleotide-mediated genome editing provides precision and function to engineered nucleases and antibiotics in plants. *Plant Physiology* 170: 1917-1928.

Shan, Q., Y. Wang, J. Li, Y. Zhang, K. Chen, Z. Liang, K. Zhang, et al. 2013. Targeted genome modification of crop plants using a CRISPR-Cas system. *Nature Biotechnology* 31: 686-688.

Shan, S., E. V. Mavrodiev, R. Li, Z. Zhang, B. A. Hauser, P. S. Soltis, D. E. Soltis, and B. Yang. 2018. Application of CRISPR/Cas9 to *Tragopogon* (Asteraceae), an evolutionary model for the study of polyploidy. *Molecular Ecology Resources* 18: 1427-1443.

Sugano, S. S., M. Shirakawa, J. Takagi, Y. Matsuda, T. Shimada, I. Hara-Nishimura, and T. Kohchi. 2014. CRISPR/Cas9-mediated targeted mutagenesis in the liverwort *Marchantia polymorpha* L. *Plant and Cell Physiology* 55: 475-481.

Svitashev, S., J. K. Young, C. Schwartz, H. Gao, S. C. Falco, and A. M. Cigan. 2015. Targeted mutagenesis, precise gene editing, and site-specific gene insertion in maize using Cas9 and guide RNA. *Plant Physiology* 169: 931-945.

Tian, S., L. Jiang, Q. Gao, J. Zhang, M. Zong, H. Zhang, Y. Ren, et al. 2017. Efficient CRISPR/Cas9-based gene knockout in watermelon. *Plant Cell Reports* 36: 399-406.

Upadhyay, S. K., J. Kumar, A. Alok, and R. Tuli. 2013. RNA-guided genome editing for target gene mutations in wheat. *G3: Genes, Genomes, Genetics* 3: 2233-2238.

Van Zeijl, A., T. A. K. Wardhani, M. S. Kalhor, L. Rutten, F. Bu, M. Hartog, S. Linders, et al. 2018. CRISPR/Cas9-mediated mutagenesis of four putative symbiosis genes of the tropical tree *Parasponia andersonii* reveals novel phenotypes. *Frontiers in Plant Science* 9: 284.

Varkonyi-Gasic, E., T. Wang, C. Voogd, S. Jeon, R. S. M. Drummond, A. P. Gleave, and A. C. Allan. 2019. Mutagenesis of kiwifruit *CENTRORADIALIS*-like genes transforms a climbing woody perennial with long juvenility and axillary flowering into a compact plant with rapid terminal flowering. *Plant Biotechnology Journal* 17: 869-880.

Wang, L., L. Wang, Q. Tan, Q. Fan, H. Zhu, Z. Hong, Z. Zhang, and D. Duanmu. 2016. Efficient inactivation of symbiotic nitrogen fixation related genes in *Lotus japonicus* using CRISPR-Cas9. *Frontiers in Plant Science* 7: 1333.

Watanabe, K., A. Kobayashi, M. Endo, K. Sage-Ono, S. Toki, and M. Ono. 2017. CRISPR/Cas9-mediated mutagenesis of the *dihydroflavonol-4-reductase-B* (*DFR-B*) locus in the Japanese morning glory *Ipomoea* (*Pharbitis*) *nil*. *Scientific Reports* 7: 10028.

Xie, K., and Y. Yang. 2013. RNA-guided genome editing in plants using a CRISPR-Cas system. *Molecular Plant* 6: 1975-1983.

Xing, H. L., L. Dong, Z. P. Wang, H. Y. Zhang, C. Y. Han, B. Liu, X. C. Wang, and Q. J. Chen. 2014. A CRISPR/Cas9 toolkit for multiplex genome editing in plants. *BMC Plant Biology* 14: 327.

Yuan, M., J. Zhu, L. Gong, L. He, C. Lee, S. Han, C. Chen, and G. He. 2019. Mutagenesis of *FAD2* genes in peanut with CRISPR/Cas9 based gene editing. *BMC Biotechnology* 19: 24.

Zhang, B., X. Yang, C. Yang, M. Li, and Y. Guo. 2016. Exploiting the CRISPR/Cas9 system for targeted genome mutagenesis in petunia. *Scientific Reports* 6: 20315.

Zhang, Z., L. Hua, A. Gupta, D. Tricoli, K. J. Edwards, B. Yang, and W. Li. 2019. Development of an *Agrobacterium*-delivered CRISPR/Cas9 system for wheat genome editing. *Plant Biotechnology Journal* 17: 1623-1635.

Zhou, H., B. Liu, D. P. Weeks, M. H. Spalding, and B. Yang. 2014. Large chromosomal deletions and heritable small genetic changes induced by CRISPR/Cas9 in rice. *Nucleic Acids Research* 42: 10903-10914.

Zhou, X., T. B. Jacobs, L. J. Xue, S. A. Harding, and C. J. Tsai. 2015. Exploiting SNPs for biallelic CRISPR mutations in the outcrossing woody perennial *Populus* reveals 4-coumarate:CoA ligase specificity and redundancy. *New Phytologist* 208: 298-301.
